# Topochemical Ring-Opening Polymerization of an Oxathianethione

**DOI:** 10.1021/jacs.5c06180

**Published:** 2025-06-14

**Authors:** Alvaro Calderón-Díaz, Liam Ordner, Maximilian G. Bernbeck, Matteo Palesati, Mark Weber, Natalie Stingelin, Will R. Gutekunst

**Affiliations:** † School of Chemistry and Biochemistry, 1372 Georgia Institute of Technology, Atlanta, Georgia 30332, United States; ‡ School of Materials Science and Engineering, Georgia Institute of Technology, Atlanta, Georgia 30332, United States

## Abstract

Single crystals of an enantiopure oxathianethione (**OTT**) were found to spontaneously convert to the corresponding polymer (**POTT**) through topochemical ring-opening polymerization (topoROP). The polymerization proceeds quantitatively and stereospecifically to give crystalline **POTT** with high molecular weights. The resulting **POTT** crystals were suitable for structure determination through X-ray crystallography to reveal polymers with right-handed helices with an antiparallel arrangement of polymer chains. Control studies support a concerted nucleophilic substitution mechanism that proceeds in the absence of radical intermediates, and the polymerization is suppressed when the monomer is randomly organized in an amorphous glass. Overall, this represents a distinct class of topochemical polymerization that opens new opportunities to prepare highly crystalline sulfur-containing materials.

## Graphical abstract

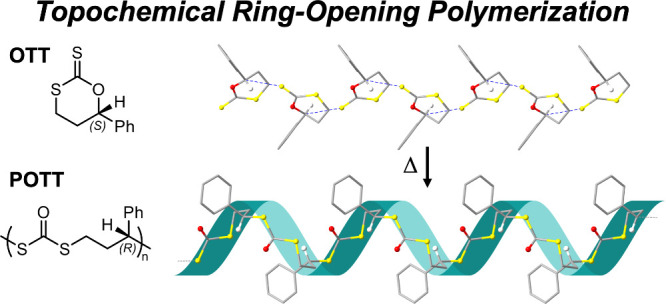

Topochemical polymerizations have found interest in research due to the completely regio- and stereoregular polymers that can be generated in the absence of solvent with high atom economy and control of polymeric architectures.^1–3^ These solid-state transformations require the appropriate arrangement of functional groups between adjacent monomers in order for a successful polymerization to occur. While initial guidelines by Schmidt necessitate sub-4.2 Å distance between reactive sites,^4,5^ this can be overcome if appropriate void space is present in the crystal for mobility.^6,7^ One of the salient features of topochemical reactions is that the product is frequently highly crystalline, and in some cases a single crystal, permitting direct characterization of the polymer structure through single crystal X-ray diffraction (SCXRD).^8^

Topochemical polymerizations were first observed in the photochemical [2 + 2] cycloadditions of diolefin derivatives by Koelsch and Gumprecht in 1958.^9^ Pioneering work by Hasegawa led to an understanding of the underlying kinetics and mechanism of this process, with later studies demonstrating the generality of topochemical [2 + 2] cycloadditions to produce a wide range of crystalline materials (Figure 1).^10–17^ Cycloadditions continue to be an actively investigated class of reaction that can further the understanding of topochemical polymerizations.^18–27^ Sureshan has recently shown the facility of thermal [3 + 2] and [4 + 2] cycloadditions when appropriately organized in the crystal lattice or xerogel assembly through polymerization of a number of sugar and peptide derivatives.^28–42^ Another major class of topochemical polymerization involves addition reactions across π-systems.^43–49^ Diyne-containing molecules have been well-documented to polymerize into conjugated poly­(diene) materials upon photoirradiation or heating in the solid-state,^50,52–56^ with polymerization also demonstrated in lipid membranes and other self-assembled arrays.^57–61^ Matsumoto’s extension of this concept to diene monomers highlights the stereospecificity of the topochemical reactions, with perfectly tritactic microstructures resulting after polymerization.^51,62–64^ Inorganic materials are also known to undergo topochemical polymerizations through distinct mechanisms, such as the ring-opening polymerization of disulfur dinitride.^65–73^ In the context of organic molecules, topochemical ring-opening polymerization (topoROP) is far less explored. Binder reported the topoROP of an *N*-carboxyanhydride monomer, though the low conversions and lack of polymer crystallinity is atypical of a topochemically mediated process.^74,75^ The serendipitous radical ring-opening polymerization of a divinyl cyclopropane in the solid-state was reported by Giorgi, Nava, and Chouraqui, though molecular packing in the solid-state prevented more than 33% conversion to polymer.^76^ In this work, we report the quantitative and stereospecific topoROP of an oxathianethione (**OTT**) monomer that proceeds through a nucleophilic substitution mechanism.^77^

**Figure 1:**
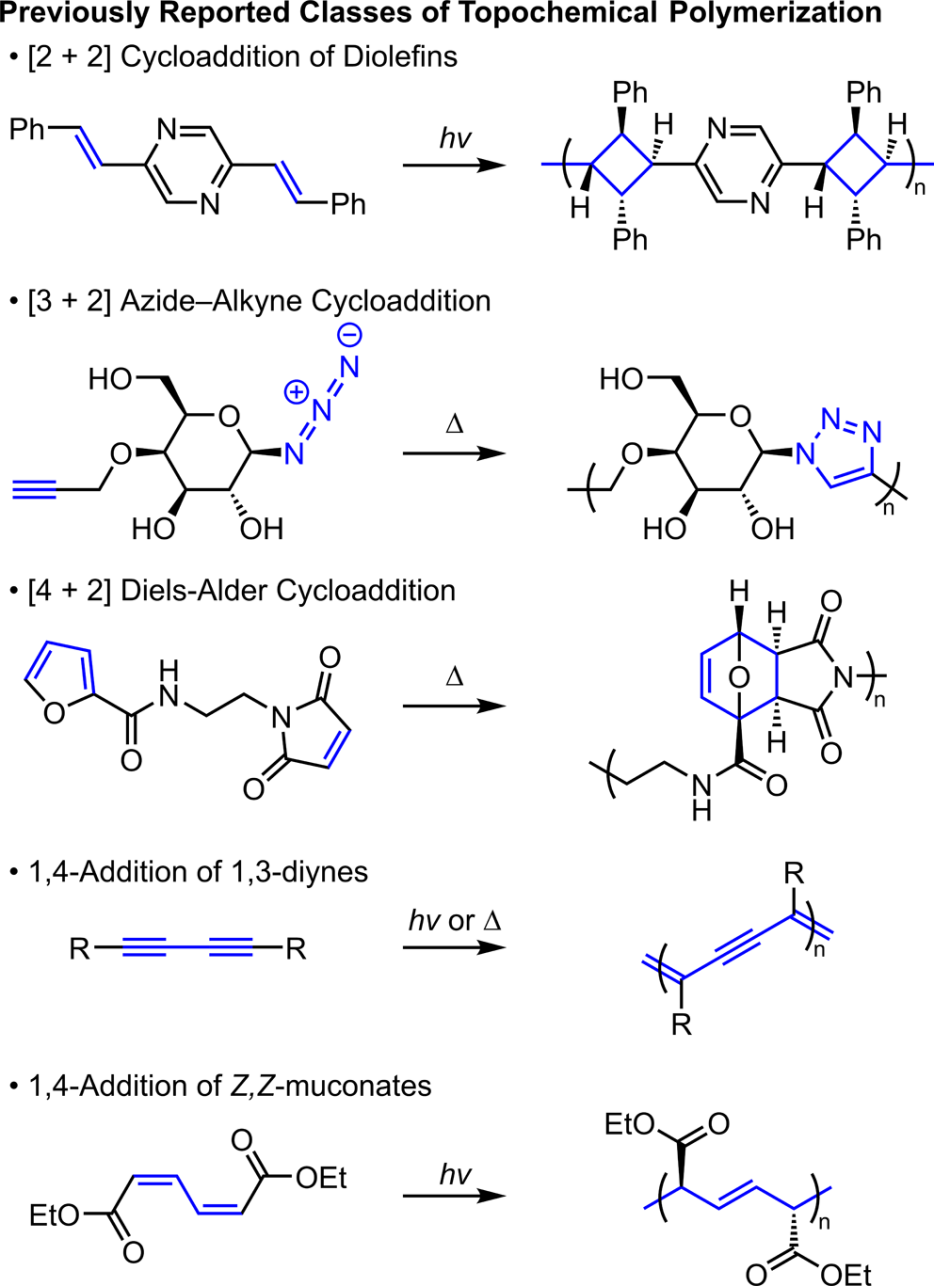
Selected representative examples of previously published topochemical polymers.^11,28,42,50,51^

Enantiopure **OTT** was prepared through a Corey-Bakshi-Shibata reduction of 3-chloropropiophenone followed by annulation of the *S*-3-chloro-phenylpropan-1-ol product with carbon disulfide under basic conditions (Figure 2A). While initially synthesized for studies in radical ring-opening copolymerization,^78–99^ it was found that quantitative polymerization occurred after storing crystalline **OTT** at ambient temperatures. The resulting polymer, **POTT**, appeared to be completely regioregular by NMR analysis, as the spectrum contained sharp sets of proton resonances and a single set of peaks in the ^13^C NMR (Figures S17 and S18). The regioregularity of **POTT** was further supported by the complete depolymerization to 4-phenyl-1,3-dithian-2-one upon treatment with 1,5,7-triazabicyclo[4.4.0]­dec-5-ene (TBD) in CH_2_Cl_2_ (Figures S20–S22).^77^ Suitable crystals for SCXRD were obtained from vapor diffusion of hexanes into CH_2_Cl_2_ solutions of **OTT**. SCXRD revealed **OTT** crystallized in the orthorhombic space group *P*2_1_2_1_2_1_ with unit cell dimensions of *a* = 7.7152(5), *b* = 9.7927(7), and *c* = 12.8333(7) Å with a volume of 969.59(11) Å^3^ (Figures S1–S3, Table S1), and the anomalous dispersion method confirmed the *S*-configuration of the stereocenter. Heating single crystals of **OTT** at 40 °C for 18 h resulted in quantitative topoROP into **POTT**, and the resulting crystals were also suitable for SCXRD analysis (Figure 2B and Figures S5–S7, Table S1). **POTT** retained the orthorhombic space group *P*2_1_2_1_2_1_ observed in **OTT**, though the unit cell dimensions showed an elongation in the *c*-direction by 21% and a decrease in the *a*- and *b*-directions by 11% and 3%, respectively. These unit cell changes can be macroscopically observed during polymerization by the geometric deformation of the single crystal, along with the formation of small cracks (Figure 2C, Video.mp4). The polymer exists in the solid-state as a right-handed single helix with a pitch length of 9.5 Å and an antiparallel arrangement of polymer chains (Figures S10 and S11).^6,100,101^ Inversion of the benzylic stereocenter to the *R*-configuration was confirmed through the anomalous dispersion method, which suggests a concerted substitution mechanism. A comparison of racemic **POTT** and *R*-**POTT** circular dichroism spectra supported the polymerization stereospecificity, as only the latter was found to be optically active with an all-positive absorbance curve below 300 nm (Figure S41).

**Figure 2:**
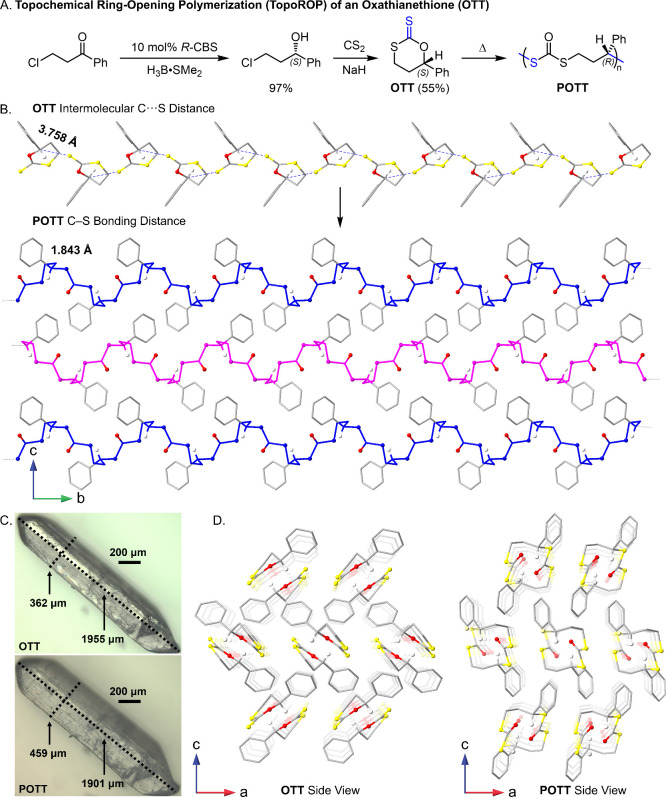
Comparison of the crystal structures of **OTT** and **POTT**. (A) Synthesis and polymerization of **OTT**. (B) Linear arrangement of **OTT** and antiparallel arrangement of **POTT**. (C) Dimensional comparison of **OTT** and **POTT** crystals. (D) Side view of **OTT** and **POTT**.

Analysis of the **OTT** crystal structure provides further insight into the polymerization process. The nucleophilic thiocarbonyl sulfur atom is in close proximity to the electrophilic benzylic carbon (C···S = 3.758 Å), which is consistent with distances observed in topochemical reactions (Figure 2B). Furthermore, the lone pair of the thiocarbonyl is well positioned to mix with the σ*_C–O_ for an S_N_2 reaction, with an S···C–O angle of 148.045°. This transition state-like orientation was previously observed by Sureshan and Watanabe in the concerted transketalization of an acetonide-protected inositol derivative.^102^ Analysis of the available void space in the **OTT** crystal (spherical probe radius: 0.6 Å) revealed that molecular motion is highly restricted with only 1.8% void space (Figure S4). This is reasonable given the organization of the monomers in a reactive arrangement, and the molecular overlay of **OTT** and **POTT** supports a rigid ring-opening polymerization process (Figure S9). The possibility of a radical mechanism was evaluated by electron paramagnetic resonance (EPR) spectroscopy, and no unpaired spin states were observed during polymerization (Figure S35). Control experiments were performed by heating **OTT** in solutions of toluene [0.24 M] or DMF [1.15 M], and no polymerization was observed (Figure S19). Collectively, these data support a topochemically mediated nucleophilic substitution mechanism for polymerization. Since this presumably proceeds through ionic chain-ends, a single initiation site that leads to multiple ring-opening events along the monomer axis is proposed (Figure S42).

Kinetic studies at 40 °C were performed by removing single crystals for analysis at different time intervals (Figure 3A, Entry 2). The polymerizations steadily increased in conversion over time, with 95% conversion reached at 6 h. Interestingly, the number-average molecular weight (*M*_*n*_) of **POTT** decreased over time from 427 kg/mol at 1 h to 289 kg/mol at 6 h. The SEC trace showed that lower *M*_*n*_ polymers emerge at later time points by the increasing shoulder at ≈10 min elution time. Matsumoto demonstrated that crystal size can control the molecular weight in topochemical polymerizations.^103^ The decrease in molecular weight at higher conversions could be the result of cracks or crystal defects forming during polymerization, which would reduce the possible path length for a given polymer chain. In agreement with Matsumoto’s findings, topoROP of microcrystalline **OTT** was heated to 60 °C for 2 h and resulted in a *M*_*n*_ of 36.6 kg/mol, which was significantly lower than that of larger crystals under analogous conditions (Figures S24 and S26).

**Figure 3:**
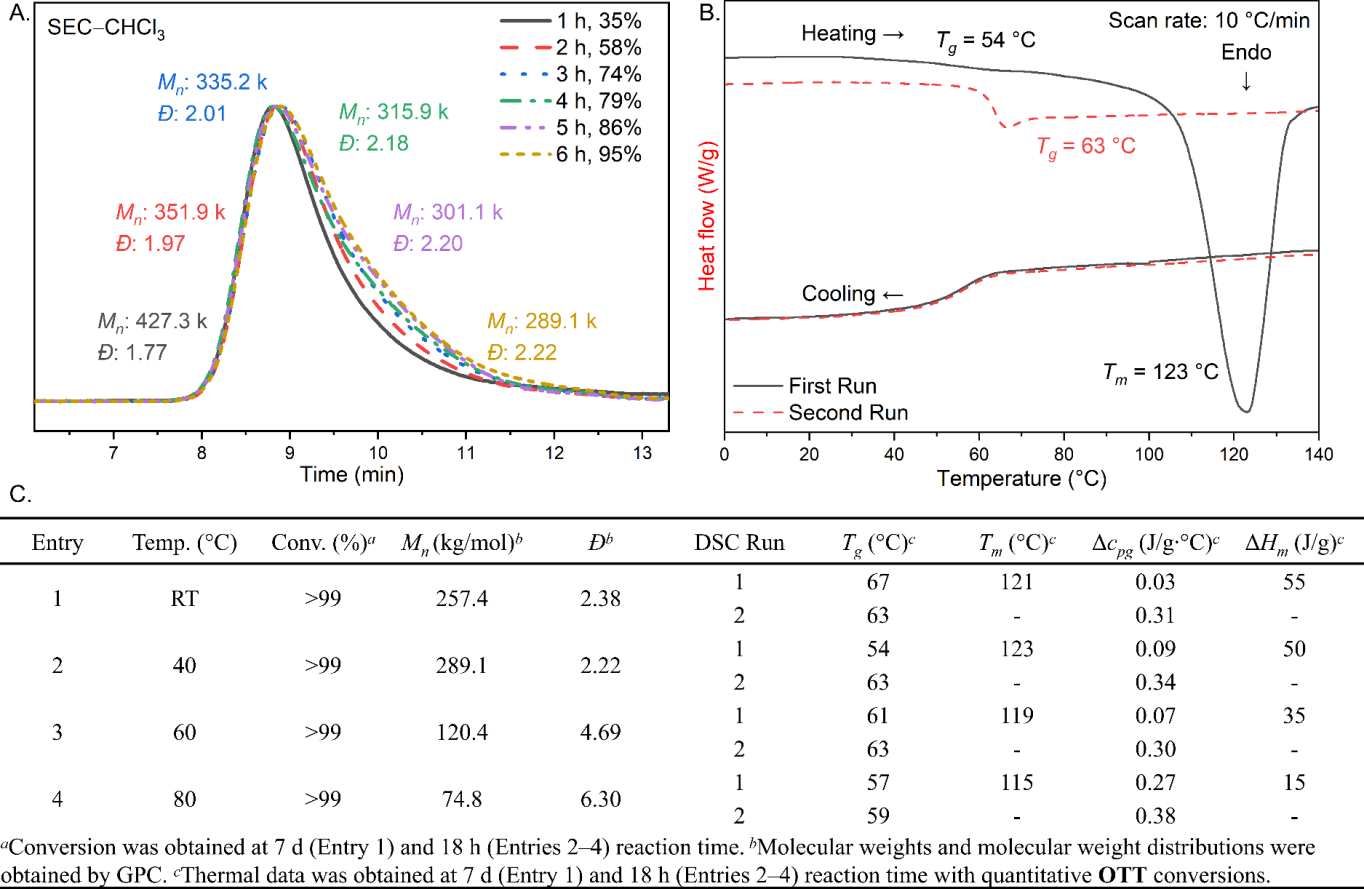
(A) GPC traces of **OTT** polymerization at 40 °C over 6 h. (B) DSC traces of **POTT** at two heating cycles. (C) Tabular summary of variable temperature topoROP of **OTT**.

In order to better understand the crystallinity of the produced **POTT**, we conducted thermal analysis experiments using a differential scanning calorimetry (DSC) run at 10 °C/min from a polymerization experiment at 18 h. A weak signal assigned to a glass transition (*T*_g_) and a notable and distinct melting endotherm were observed on the first heating cycle at 54 °C (Δ*c*_*pg*_ = 0.09 J/g·°C) and 123 °C (enthalpy of melting, Δ*H*_*m*_ = 50 J/g), respectively (Figure 3B). This is in agreement with a highly crystalline polymer with minimal amorphous content. No crystallization was observed upon cooling, and a drastically more intense glass transition signal was present in the second heating cycle (*T*_g_ = 63 °C, Δ*c*_*pg*_ = 0.34 J/g·°C). Despite the regio- and stereoregularity of **POTT**, attempts to anneal the polymer for extended times resulted in only amorphous material.

Additional polymerization experiments were performed at room temperature, 60 °C, and 80 °C (Figures S23–S25). Polymerization rates increased as temperature increased, with the 60 and 80 °C experiments reaching full conversion at 120 and 30 min, respectively (Figures S27–S30). Interestingly, elevated temperatures led to polymers of lower *M*_*n*_ and decreased crystallinity (Figure 3, Entries 1–4). As seen in the experiment at 40 °C, a gradual reduction in the *M*_*n*_ was observed as the polymerizations progressed. Faster polymerizations led to increased defects in the crystal lattice, as geometric distortion of the crystals is evident in experiments near or above the *T*_g_. The reduction in crystallinity at higher temperatures can be seen in the melting enthalpies (Δ*H*_*m*_ *)* measured by DSC (Figures S36–S38). Δ*H*_*m*_ decreased from 55 J/g for **POTT** polymerized at room temperature to 15 J/g for **POTT** polymerized at 80 °C.

Fast scanning calorimetry (FSC)^93,94^ was employed to measure the melting temperature of the small molecule **OTT** at heating rates of 50 °C/s—information that is unobtainable through classical methods due to concomitant polymerization. The rapid FSC scan rates (50–4,000 °C/s) permitted the measurement of a very sharp *T*_*m*_ for **OTT** at 140 °C without detectable polymerization (Figure 4). Subsequent cooling at 50 °C/s produced an amorphous monomer glass that featured a *T*_g_ (onset) around −10 °C upon heating (Figure 4).

**Figure 4:**
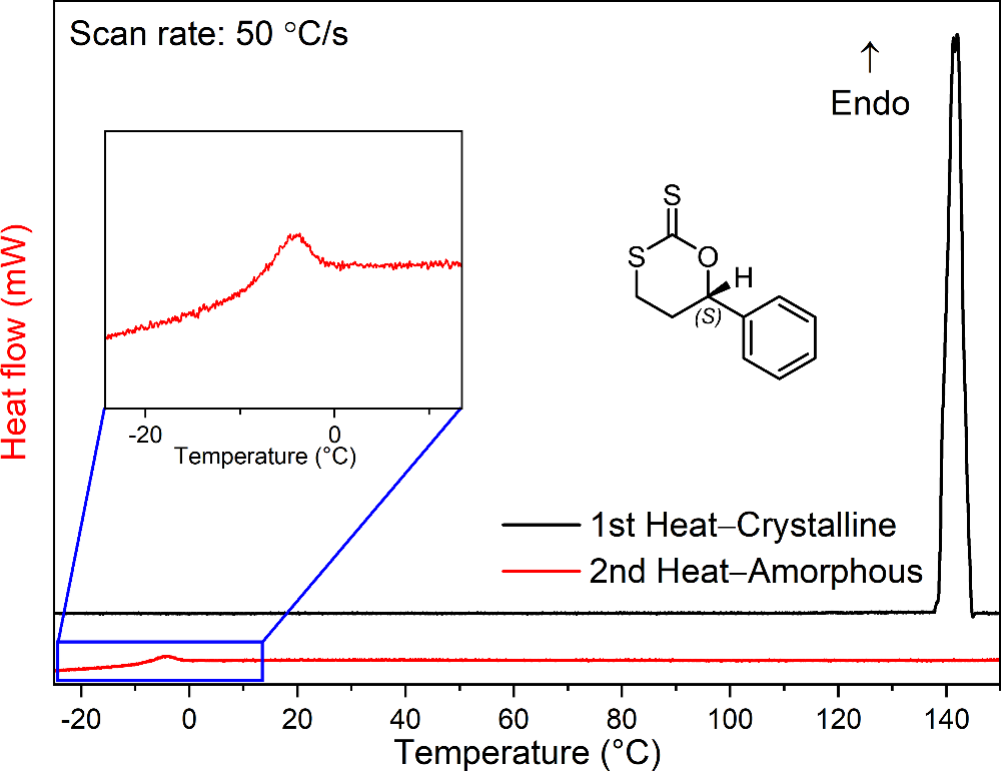
FSC scans of crystalline and amorphous **OTT**.

Further thermal analysis was performed comparing **OTT** glasses that were aged for 30 min at temperatures ranging from –90 to 100 °C vs unaged glasses (i.e., rapidly cooled materials that were then heated directly at 4,000 °C/s without an aging step; Figure S39). Three specific regimes can be identified, allowing us to determine the rate-independent *T*_g_ ^104,105^ and, importantly, the onset temperature of polymerization.

In the first regime (aging at –90 to 10 °C), the heat flow rate of the aged samples is in a specific temperature range in excess of that measured for the unaged references, resulting in clear enthalpic overshoots,^104,105^ red highlighted areas in Figure S39. A linear extrapolation can be performed to determine the temperature at which the excess heat flow is zero, which defines the rate-independent *T*_g_ (here: ≈10 °C).

The second regime is observed for aging temperatures of 10 to 70 °C, that is at aging temperatures where no enthalpic overshoot is observed anymore (i.e., aging above *T*_g_). A clear step change in heat flow, characteristic for a glass transition (onset ≈10 °C), is recorded for both aged material and the unaged reference. In the third regime, this feature (step change in heat flow) shifts gradually to higher temperatures indicating an increase in glass transition — an unambiguous sign for polymerization to occur (an increase in molecular weight will lead to an increase in glass transition temperature). These observations, hence, further support the topochemical nature of the polymerization, as the amorphous monomer is unreactive at temperatures in which the crystalline monomer rapidly polymerizes.

In conclusion, the topochemical ring-opening polymerization of the oxathianethione **OTT** has been presented. The polymerization proceeds stereospecifically to give highly crystalline **POTT** in quantitative yields. SCXRD of **POTT** revealed the molecular structure to be a right-handed helix with an antiparallel arrangement of polymer chains in the crystal lattice. High molecular weights were observed, which decreased as a function of crystal quality and size. Complete inversion of the benzylic stereocenter during polymerization supports a nucleophilic substitution mechanism of ring-opening, which is suppressed when **OTT** is heated as an amorphous glass. Given the ability to depolymerize **POTT**, these precision sulfur-containing polymers may find application in degradable materials for 3D printing, as recently demonstrated in other sulfur-based ROP systems.^106–108^ Future work will explore the generality of cyclic thiocarbonyl compounds in topochemical ring-opening polymerization for the preparation of precision sulfur-containing materials.

## Supplementary Material





## Notes

Full experimental details, crystallographic data, ^1^H and ^13^C NMR spectra of **OTT** and **POTT**, EPR, and SEC traces of **OTT** (PDF)
Polymerization video for **OTT** (MP4)
