# Supplementary material for: Topochemical Ring-Opening Polymerization of an Oxathianethione
Source: J Am Chem Soc. 2025 Jun 14;147(25):21331–8. doi: 10.1021/jacs.5c06180 (PMC12203593; doi:10.1021/jacs.5c06180)
Supplement: Supplementary file 1 [file ja5c06180_si_001.pdf]

Supporting Information

Topochemical Ring-Opening Polymerization of an Oxathianethione

Alvaro Calderón-Díaz,[†] Liam Ordner,[†] Maximilian G. Bernbeck,[†] Matteo Palesati,[‡] Mark Weber,[‡] Natalie Stingelin,[‡] and Will R. Gutekunst*[,†,‡]

[†] School of Chemistry and Biochemistry, Georgia Institute of Technology, 901 Atlantic Drive NW, Atlanta, Georgia 30332, United States.

[‡] School of Materials Science and Engineering, Georgia Institute of Technology, Atlanta, Georgia 30332, United States.

*E-mail: willgute@gatech.edu

Table of Contents

General Methods S3

Scheme S1: CBS reduction of (*S*)-3-chloro-1-phenylpropan-1-ol S4

Synthesis of (*S*)-3-chloro-1-phenylpropan-1-ol (**I**) S4

Scheme S2: Synthesis of **OTT** S4

Synthesis of (*S*)-6-phenyl-1,3-oxathiane-2-thione (**OTT**) S4

General Procedure for the Preparation of **OTT** Crystals S5

Topochemical Polymerization of **OTT** S5

Scheme S3: Depolymerization of **POTT** S6

Depolymerization of **POTT** S6

X-ray Crystallography S6

Differential Scanning Calorimetry S7

Flash Scanning Calorimetry S7

Preparation of Microcrystals S7

Figure S1–S11: Crystallographic structure representations S8

Table S1: Crystal data and refinement details for **OTT** and **POTT** S18

Figures S12–S14: Crystal images of **OTT** and **POTT** S19

Figures S15–S22: NMR spectra of **OTT** and **POTT** S20

Figures S23–S34: SEC traces and conversion plots for variable temperature **POTT** S24

Figure S35: EPR spectrum of **POTT** S30

Figures S36–S39: DSC and FSC traces of variable temperature **POTT** S30
Figures S40: TGA of **POTT** S33
Figures S41: CD spectrum of racemic and enantiopure **POTT** S33
Figure S42: Proposed mechanism for the topochemical ring-opening of **OTT** S34
References S35

General methods. All synthetic procedures involving air- and moisture-sensitive compounds were carried out using Schlenk technique under an atmosphere of nitrogen. Glassware was heat-sealed with a heat gun under vacuum.

Solvents: Prior to use, THF and CH_2Cl_2 were purified using a PPT Purification System. For non-inert manipulations, THF, CH_2Cl_2, diethyl ether, MeOH, ethyl acetate, hexanes (mixture of isomers) were used as received without further purification.

Deuterated solvents: $CDCl_3$ (Cambridge Isotope Laboratories, Inc., D, 99.8 % v/v) was used as received.

Reactants: 3-Chloropropiophenone (TCI America, >96.0%), borane dimethyl sulfide complex (Sigma-Aldrich, neat), carbon disulfide (Supelco, ≥99.9%), (*R*)-(+)-2-methyl-CBS-oxazaborolidine (Sigma-Aldrich, ≥90%), sodium hydride (TCI America, 60% dispersion in paraffin liquid), 1,5,7-triazabicyclo[4.4.0]dec-5-ene (TCI America, >98.0%), trifluoroacetic acid (TCI America, >99.0%).

NMR measurements were recorded on a Bruker Avance III 400 and 500 spectrometer at ambient probe temperatures unless otherwise noted. $^{13}C\{^{1}H\}$ NMR resonances were obtained with proton broadband decoupling and referenced to the solvent signals of $CDCl_3$ at 77.2 (^{1}H NMR: 7.26 ($CHCl_3$)). $^{13}C\{^{1}H\}$ NMR assignments are based on DEPT 135, and the following 2D experiments: COSY, NOESY, ROESY, HSQC, and HMBC. Mass-spectrometric analyses were performed on an Agilent Technologies 1260 Infinity II/6120 Quadrupole time of flight MS system (low resolution ESI). IR spectra were measured on a Shimadzu IRAffinity-1 FT-IR Spectrometer equipped with a Universal ATR Sampling Accessory. UV/Vis spectra of solutions of **OTT** and **POTT** in CH_2Cl_2 were measured with a Cary 5000 spectrometer. Polymer analyses were performed using a Tosoh EcoSEC HLC 8320 GPC with a TSKgel SuperHZ-L column at a 0.45 mL/min flow rate of (eluted using $CHCl_3$ containing 0.25% NEt_3). All number-average molecular weights and dispersity values were calculated from refractive index chromatograms using PStQuick Mp-M polystyrene standards. Differential Scanning Calorimetry (DSC) analyses were measured at a 10 °C/min ramp rate on a TA Instruments DSC250 under high purity nitrogen equipped with a Refrigerated Cooling System (RSC90) chiller. All DSC thermal analysis values were calculated using the Trios Data Analysis software. Flash Scanning Calorimetry (FSC) analyses were measured on a Mettler Toledo Flash DSC 1 under high purity nitrogen equipped with a Huber TC100 chiller. All FDSC thermal analysis values were calculated using the STAR-E software and OriginPro. Crystal were viewed on an Olympus BX51 polarizing microscope equipped with a QImaging MicroPublisher 5.0 RTV camera and Linkam LTSE 420 Heating and Freezing stage system. Crystal images and videos were recorded using the Linksys 32 software. EPR measurements were recorded on a Bruker ELEXSYS-E500 spectrometer.

Circular dichroism (CD) was measured on a JASCO J-810 instrument with polymer solution concentration of 5.39 × 10^{-4} mg/μL in CH_2Cl_2.

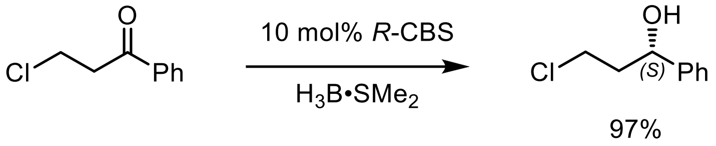

Scheme S1. CBS reduction of (*S*)-3-chloro-1-phenylpropan-1-ol.

Synthesis of (*S*)-3-chloro-1-phenylpropan-1-ol (1)

Under inert conditions, *R*-CBS (330.19 mg, 1.19 mmol) was dissolved in THF (10.0 mL) and chilled (0 °C) for 15 min followed by the dropwise addition of $H_3B{\cdot}SMe_2$ (1.2 mL, 905.02 mg, 11.91 mmol). After an additional 15 min stir at 0 °C, a solution of 3-chloropropiophenone (2.01 g, 11.91 mmol) in THF (45 mL) was added dropwise to the chilled mixture and stirred for 15 min. The solution was allowed to warm to room temperature and stirred for an additional 1 h. The reaction was quenched with MeOH (30 mL) and diluted with ethyl acetate (200 mL). The organic phase was subsequently washed with acidic water (1.0 M, 3 × 70 mL), water (3 × 70 mL), brine (70 mL), dried over anhydrous sodium sulfate, filtered, and reduced to dryness. The resulting residue was dried under vacuum for 3 h. Yield: 1.964 mg (11.51 mol, 97%)

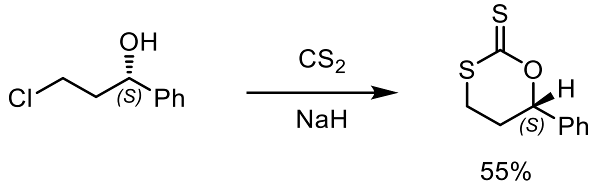

Scheme S2. Synthesis of **OTT**.

Synthesis of (*S*)-6-phenyl-1,3-oxathiane-2-thione (OTT)

NaH (143.51 mg, 5.98 mmol) was suspended in THF (8.0 mL) and chilled (0 °C) for 10 min followed by the dropwise addition of a solution of CS_2 (0.76 mL, 931.70 mg, 12.24 mmol) in THF (8.0 mL). A solution of (*S*)-3-chloro-1-phenylpropan-1-ol (416.43 mg, 2.44 mmol) in THF (8.0 mL) was subsequently added dropwise to the NaH/CS_2 mixture over a period of 5 min and stirred for 10 min. The mixture was allowed to warm to room temperature and stirred for an additional 2 h. The yellow mixture was cooled (0 °C), quenched with saturated ammonium chloride (aq), and stirred for 30 min. The reaction was then diluted with water (20 mL) and CH_2Cl_2 (50 mL). The organic phase was separated and washed with water (2 × 30 mL), brine (30 mL), dried over anhydrous sodium sulfate, filtered, and reduced to dryness. The residue was redissolved in fresh CH_2Cl_2 (6.0 mL) and recrystallized from vapor diffusion with hexanes at (–3 °C). The isolated pale-yellow crystals were then dried under vacuum for 30 min. Yield: 282.27 mg (1.34 mol, 55%); T_m : ≈138 °C. ^{1}H NMR (500.1 MHz, $CDCl_3$): δ 2.34–2.41 (m, 1 H; CH_2, CHC$\boldsymbol{H}_2$), 2.53 (dtd, $^3J_{H,H}$

= 14.8, 4.8, 2.1 Hz, 1 H; CH_2, CHC***H***$_2$), 3.01 (dt, $^3J_{H,H}$ = 12.0, 4.7 Hz, 1 H; CH_2, SC***H***$_2$), 3.27 (td, $^3J_{H,H}$ = 11.6, 4.9 Hz, 1 H; CH_2, SC***H***$_2$), 5.49 (dd, $^3J_{H,H}$ = 9.5, 1.8 Hz, 1 H; CH), 7.37–7.44 (m, 5 H; CH, *o*-, *m*-, and *p*-Ph). ^{13}C NMR (125.8 MHz, $CDCl_3$): δ 27.9 (CH_2, CH***C***H$_2$), 30.2 (CH_2, S***C***H$_2$), 85.1 (CH), 126.3, 129.1, 129.3 (CH, *o*-, *m*-, and *p*-Ph), 137.8 (C, *ipso*-Ph), 209.2 (COS_2). MS (ESI(+)): *m/z* (relative intensity) 113 (100) $[M + Na]^{2+}$, 210 (9) $[M]^+$. IR (neat, cm-1): $\tilde{\nu}$ = 3063, 3036, 2943, 2927 (w, *v*(C–H)), 1645 (w, *v*(C=C)), 1493 (m), 1456 (m), 1441, 1420 (w), 1375 (w), 1341 (m), 1333, 1314 (m), 1302 (m), 1279 (m), 1221 (s), 1206 (s), 1177, 1167 (s), 1153 (s), 1115, 1109, 1099, 1086, 1067 (s), 1043 (s), 1008 (m), 988, 980, 960 (s), 939, 926 (w), 918 (m), 899 (m), 885, 876, 872, 862, 849 (m), 841 (m), 826, 810, 806 (m), 799, 785, 766 (s), 752, 739, 729, 719, 698 (s), 687, 675, 667, 654, 650, 644, 633, 631, 627, 619, 610. UV–Vis (CH_2Cl_2): λ [nm] (ε [$L \cdot mol^{-1} \cdot cm^{-1}$]) 298, 1.06×10^4.

General Procedure for the Preparation of OTT Crystals

Using an 8 mL vial, crystals of **OTT** (25.46 mg) were dissolved in CH_2Cl_2 (0.3 mL). The open capped vial was then placed in a 20 mL vial containing hexanes (5.0 mL). The 20 mL vial was capped, wrapped with parafilm, and stored at –3 °C for 18 h. The supernatant was then decanted and the solvated crystal were transferred to clean vials. The crystals were then dried under vacuum at 0 °C for 15 min. These crystals were isolated and immediately used for the topochemical polymerization of **OTT**.

Topochemical Polymerization of OTT

Freshly isolated crystals of **OTT** (20.3 mg, 0.12 mmol) were placed in a 4 mL vial and heated to target temperature. Yields after reaction completion were quantitative and determined by ^{1}H NMR spectroscopy. These crystals were immediately used for crystallographic and DSC studies to minimize aging. ^{1}H NMR (500.1 MHz, $CDCl_3$): δ 2.23 (q, $^3J_{H,H}$ = 7.5 Hz, 2 H; CH_2, CHC***H***$_2$), 2.87 (t, $^3J_{H,H}$ = 7.4 Hz, 2 H; CH_2, SC***H***$_2$), 4.70 (t, $^3J_{H,H}$ = 7.8 Hz, 1 H; CH), 7.22–7.25 (m, 3 H; CH, *o*-Ph and *p*-Ph), 7.28–7.31 (m, 2 H; CH, *m*-Ph). ^{13}C NMR (125.8 MHz, $CDCl_3$): δ 28.5 (CH_2, S***C***H$_2$), 36.2 (CH_2, CH***C***H$_2$), 48.7 (CH), 127.8 (CH, *o*-Ph), 128.1 (CH, *p*-Ph), 129.1 (CH, *m*-Ph), 139.9 (C, *ipso*-Ph), 188.0 (COS_2). IR (neat, cm-1): $\tilde{\nu}$ = 3028, 2938, 2932, 2920 (w, *v*(C–H)), 1714 (w), 1678, 1672, 1643 (s, *v* (C=O)), 1602, 1581, 1564, 1558, 1500, 1489 (m), 1472 (w) 1452 (m), 1439, 1433 (w), 1422, 1402 (m), 1377, 1366, 1348 (m), 1341, 1333, 1317, 1313 (w), 1302 (w), 1294 (w), 1273, 1267 (w), 1224 (m), 1207 (m), 1179, 1170 (m), 1157, 1150, 1138, 1126, 1117, 1109, 1092, 1072, 1065 (m), 1043, 1030, 1011, 1005, 991, 984, 980, 962 (w), 949, 939 (w), 926, 924, 918 (w), 856 (s and br), 837, 804, 795, 777, 770, 750 (s), 733, 727, 708 (m), 696 (s), 679, 671, 664, 654, 648, 642, 635, 627, 615, 608, 604. UV–Vis (CH_2Cl_2): λ [nm] (Abs [au]) 253, 0.55.

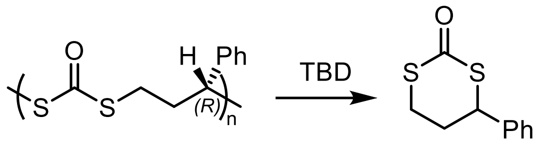

Scheme S3. Depolymerization of **POTT**.

Depolymerization of POTT

POTT (28.35 mg, 0.15 mmol, M_n = 185.2 kg/mol, $Đ$ = 3.43, DP = 880) was dissolved in CH_2Cl_2 (10 mL) and stirred for 5 min. A solution of 1,5,7-triazabicyclo[4.4.0]dec-5-ene (9 µL, 0.41 mg, 2.97 mmol, 20 equiv. relative to number of moles of polymer chains based on M_n measured by SEC) in CH_2Cl_2 (4.6 mg/mL) was subsequently added to the **POTT** suspension and stirred for 18 h at room temperature. The depolymerization reaction was quenched using trifluoroacetic acid (3 drops). Depolymerization conversion was determined to be quantitative by crude ^{1}H NMR. The solution was then reduced to dryness. The resulting residue was purified by preparative thin layer chromatography and eluted with hexanes/EtOAc (8:2 v/v) to give a colorless oil. Yield: 20.30 mg (0.10 mmol, 72%); ^{1}H NMR (500.1 MHz, $CDCl_3$): δ 2.44 (dtd, $^3J_{H,H}$ = 14.1, 10.3, 3.5 Hz, 1 H; CH_2, CHC***H***$_2$), 2.66 (ddt, $^3J_{H,H}$ = 14.0, 6.9, 3.5 Hz, 1 H; CH_2, CHC***H***$_2$), 3.32 (ddd, $^3J_{H,H}$ = 13.4, 6.7, 3.5 Hz, 1 H; CH_2, SC***H***$_2$), 3.43 (ddd, $^3J_{H,H}$ = 13.3, 9.8, 3.4 Hz, 1 H; CH_2, SC***H***$_2$), 4.72 (dd, $^3J_{H,H}$ = 10.9, 3.5 Hz, 1 H; CH), 7.33–7.41 (m, 5 H; CH, *o*-, *m*-, and *p*-Ph). ^{13}C NMR (125.8 MHz, $CDCl_3$): δ 30.8–30.9 (CH_2, CH***C***H_2 and S***C***H_2), 51.6 (CH), 127.9 (CH, *o*-Ph), 128.7, 129.3 (CH, *m*-, and *p*-Ph), 139.0 (C, *ipso*-Ph), 189.9 (COS_2). MS (ESI(+)): *m/z* (relative intensity) 117 (100) $[M + Na]^{2+}$, 211 (36) $[M + H]^+$. IR (neat, cm-1): $\tilde{\nu}$ = 3177, 3057, 3028, 2934, 2920 (w, *ν*(C–H)), 2849, 1593 (s, *ν*(C=O)), 1557, 1489 (m), 1452 (m), 1294 (w), 1277 (w), 1260 (w), 1238 (w), 1184 (w), 1157 (w), 1144 (w), 1076 (w), 1059, 1206 (s), 1030, 1016 (w), 1001, 986, 961, 943, 918, 887 (s), 795 (m), 758 (s), 696 (s), 665, 627, 611 (m), 926 (w), 918 (m).

X-ray Crystallography

Single crystals of **OTT** were obtained by allowing hexane vapor to slowly diffuse into a CH_2Cl_2 solution at –3 °C pale yellow elongated rectangular prisms. These crystals were subsequently measured by X-ray diffraction after polymerization (**POTT**). X-ray data for **OTT** and **POTT** were collected on a Bruker D8 VENTURE dual wavelength four-circle diffractometer with a microfocus sealed X-ray tube (MoKa radiation, λ = 0.71073 Å) by using ω and φ scans after shock-cooling a single crystal at 100(1) K (Table S1). Data was collected on a Bruker PHOTON III detector. All data were integrated with SAINT V8.B40[1] in APEX4 and a multiscan absorption correction and scaling was performed using SADABS[2] as implemented in APEX4. Cell parameters were obtained and refined with 54449 (3233 unique, **OTT**) and 54535 (2245 unique, **POTT**) reflections. The structures were solved with the ShelXT structure solution program using the Intrinsic Phasing solution method and refined by full-matrix least-squares methods

against F^2 using SHELXL-2014[3] and Olex2 1.5[4] as the graphical interface. All hydrogen atoms were refined isotropic on calculated positions using a riding model with their U_{iso} values constrained to 1.5 times the U_{eq} of their pivot atoms for terminal sp^3 carbon atoms and 1.2 times for all other carbon atoms. Non-hydrogen atoms were refined with anisotropic thermal parameters. Absolute structure determination was determined by anomalous dispersion. This report and the CIF file were generated using FinalCif.[5] Crystallographic data for the structures reported herein have been deposited with the Cambridge Crystallographic Data Centre.[6] Deposition Number(s) CCDC 2383720 (for **OTT**) and 2383721 (for **POTT**) contain(s) the supplementary crystallographic data for this paper. These data are provided free of charge by the joint Cambridge Crystallographic Data Centre and Fachinformationszentrum Karlsruhe Access Structures service www.ccdc.cam.ac.uk/structures. Void space analyses were performed using Mercury 2024.3.1. (spherical probe size: 0.6 Å, approximate grid spacing: 0.3 Å).[7]

Differential Scanning Calorimetry

POTT crystalline material for differential scanning calorimetry (DSC) was prepared in a TA Instruments TZero pan and sealed with TZero lids. A sealed TZero pan and TZero lid was used as a reference standard. Measurements were conducted at heating/cooling rates of 10 °C/min between –20 °C and 150 °C, under nitrogen. Two cycles were recorded.

Flash Scanning Calorimetry

OTT crystals were deposited onto Mettler Toledo Standard MultiSTAR UFS 1 chips and measurements were performed under nitrogen using a Mettler Toledo Flash DSC 1 equipped with a Huber TC100 cooler to control the temperature. In a first set of experiments, the samples were measured in multiple, successive heating and cooling cycles as in standard DSC but at heating/cooling rates of 50, 100, and 4000 °C/s to prevent polymerization and, hence, allowing us to determine a melting temperature, T_m, and glass transition temperature, T_g. In addition to these measurements, physical aging experiments were performed.[8–9] Figure S34, top panel, depicts the aging method used here following procedures in literature (Refs. 8–9). Samples were initially heated to erase any thermal history and immediately cooled to the aging temperature, T_a, at a rate of –4000 °C/s. At this temperature, samples were aged for 30 min and subsequently cooled at –4000 °C/s prior to rapid heating at +4000 °C/s. This scan delivered the heat flow rate of aged samples. Finally, samples were cooled at –4000 °C/s to –90 °C and immediately heated again at +4000 °C/s to obtain the reference thermogram for unaged samples.

Preparation of Microcrystals

Microcrystals were prepared in analogy to a literature procedure, with modifications.[10] **OTT** (53.7 mg. 0.26 mmol) was dissolved in benzene (5 mL) and cooled in liquid nitrogen. The frozen solution of benzene was placed under vacuum for sublimation and warmed to 0 °C for 4 h.

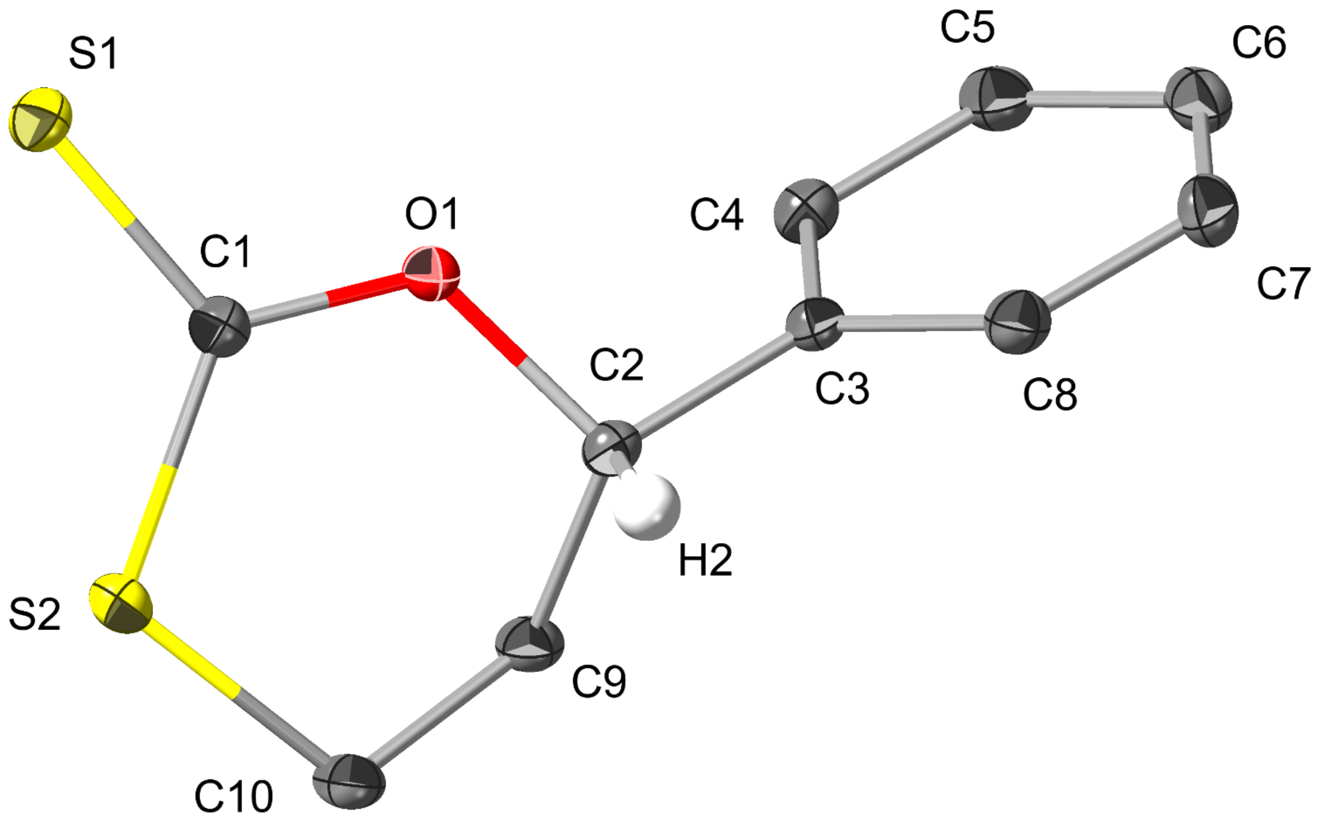

Figure S1. Molecular structure of **OTT** showing ellipsoids with anisotropic displacement factors at 50% probability. Hydrogen atoms have been omitted for clarity. Selected interatomic distances (Å), bond angles (deg), and torsion angles (deg): S1–C1 1.6517(13), S2–C1 1.7300(13), S2–C10 1.8299(14), O1–C1 1.3284(15), O1–C2 1.4818(16), C2–C9 1.5192(18), C2–C3 1.5012(18), S1–C1–S2 118.02(7), S1–C1–O1 118.78(10), S2–C1–O1 123.20(10), C1–S2–C10 106.07(6), C1–O1–C2 121.70(10), O1–C2–C3 104.95(10), O1–C2–C9 109.57(11), C9–C2–C3 114.90(11), S1–C1–O1–C2 168.77(9), S1–C1–S2–C10 172.57(7), C1–O1–C2–C9 51.15(15), C1–O1–C2–C3 175.04(11), C1–S2–C10–C9 –13.63(11), O1–C2–C9–C10 –71.26(13), S2–C10–C9–C2 50.91(14), O1–C2–C3–C4 –58.73(15).

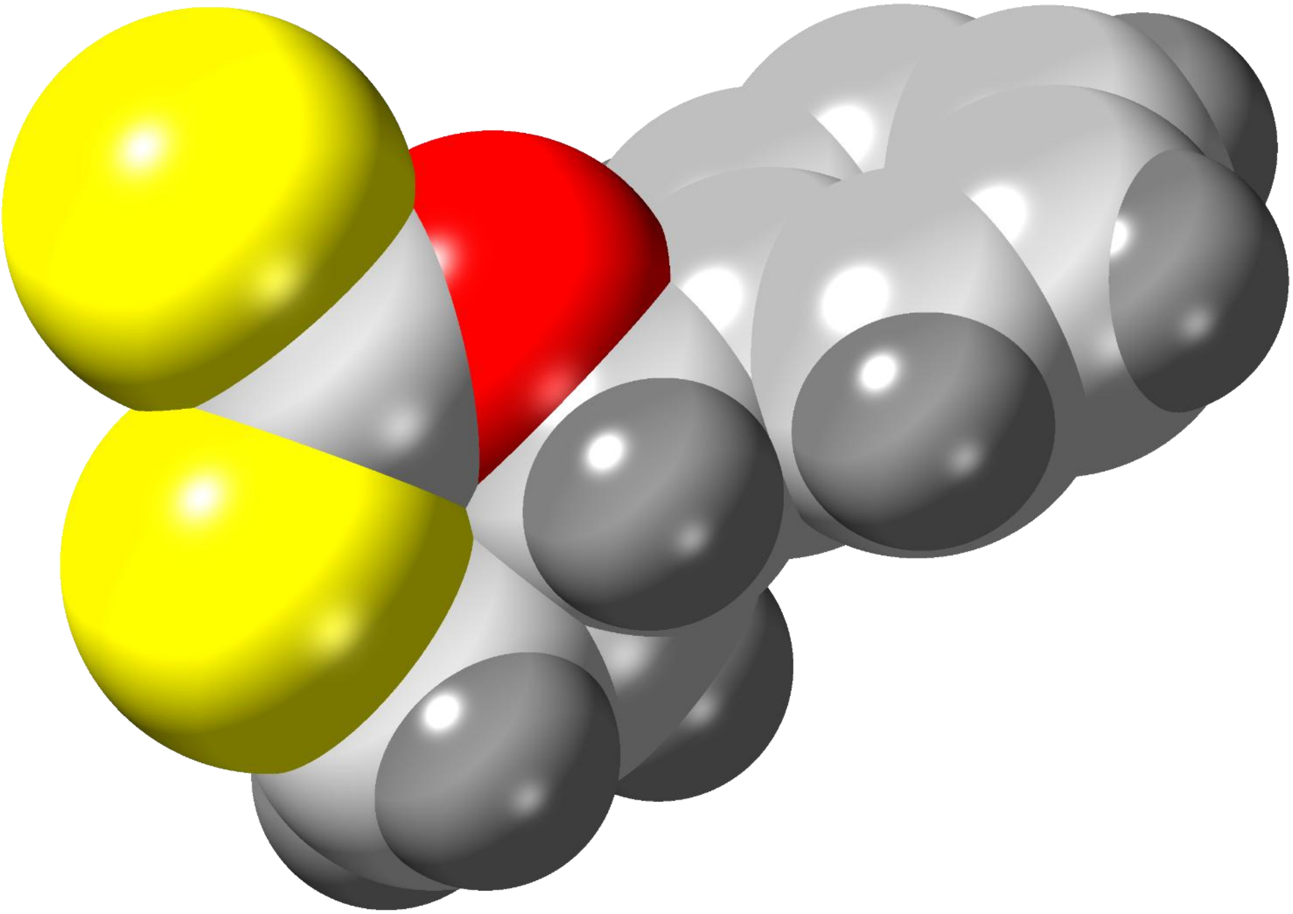

Figure S2. Space filling representation of the molecular structure of **OTT**.

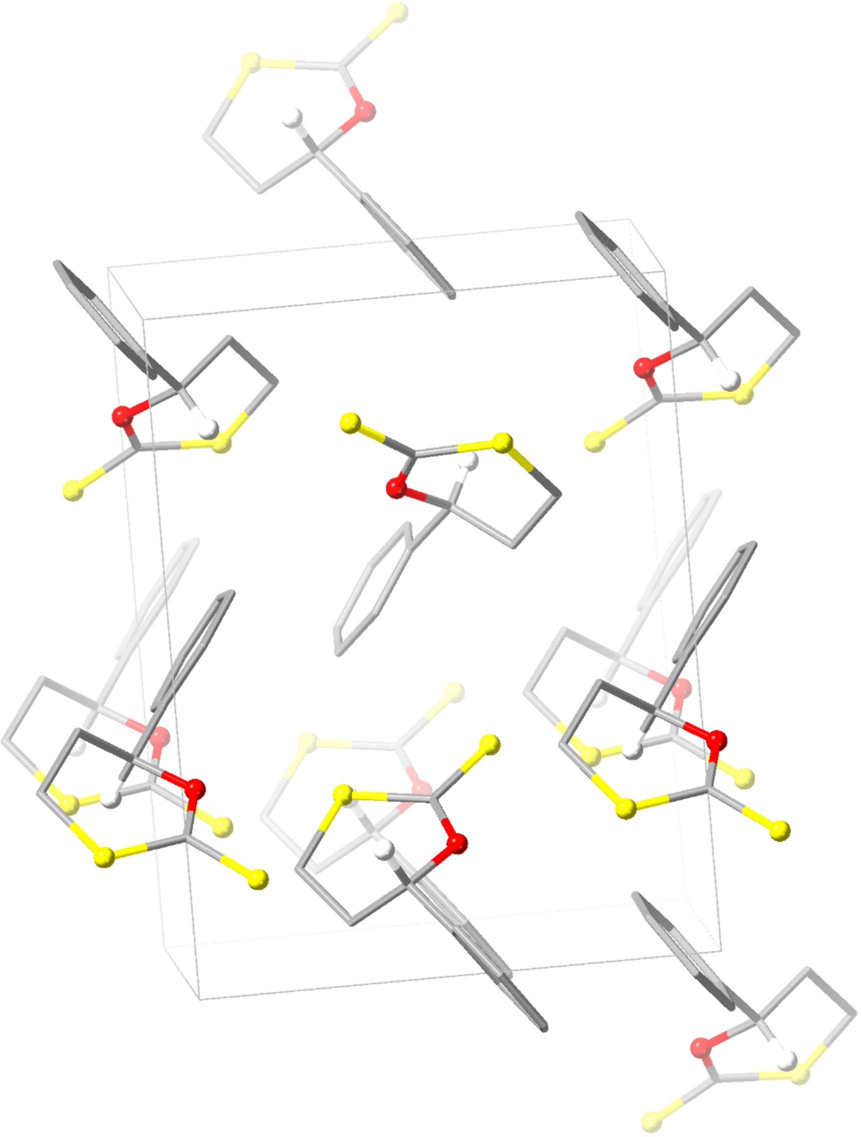

Figure S3. Crystal packing diagram of the unit cell of **OTT**. Hydrogen atoms have been omitted for clarity.

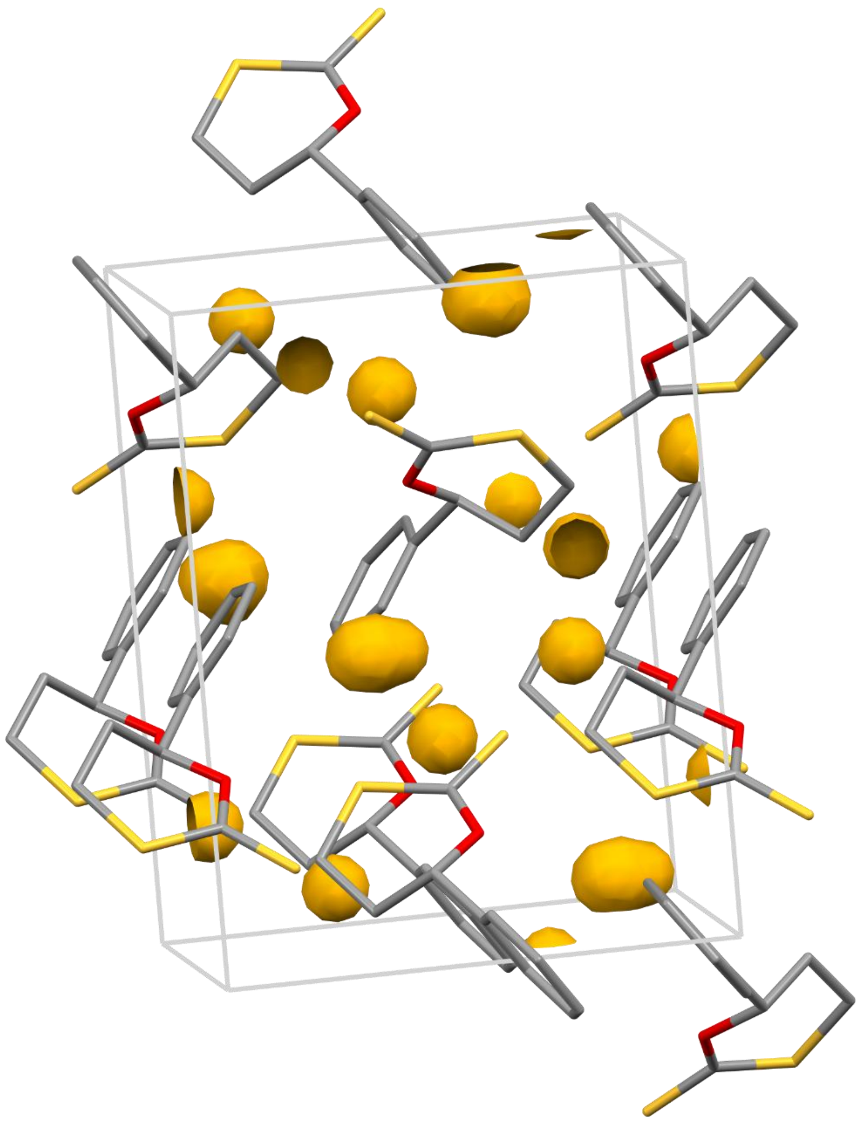

Figure S4. Crystal packing diagram of the unit cell of **OTT** showing void spaces (spherical probe radius: 0.6 Å). Hydrogen atoms have been omitted for clarity.

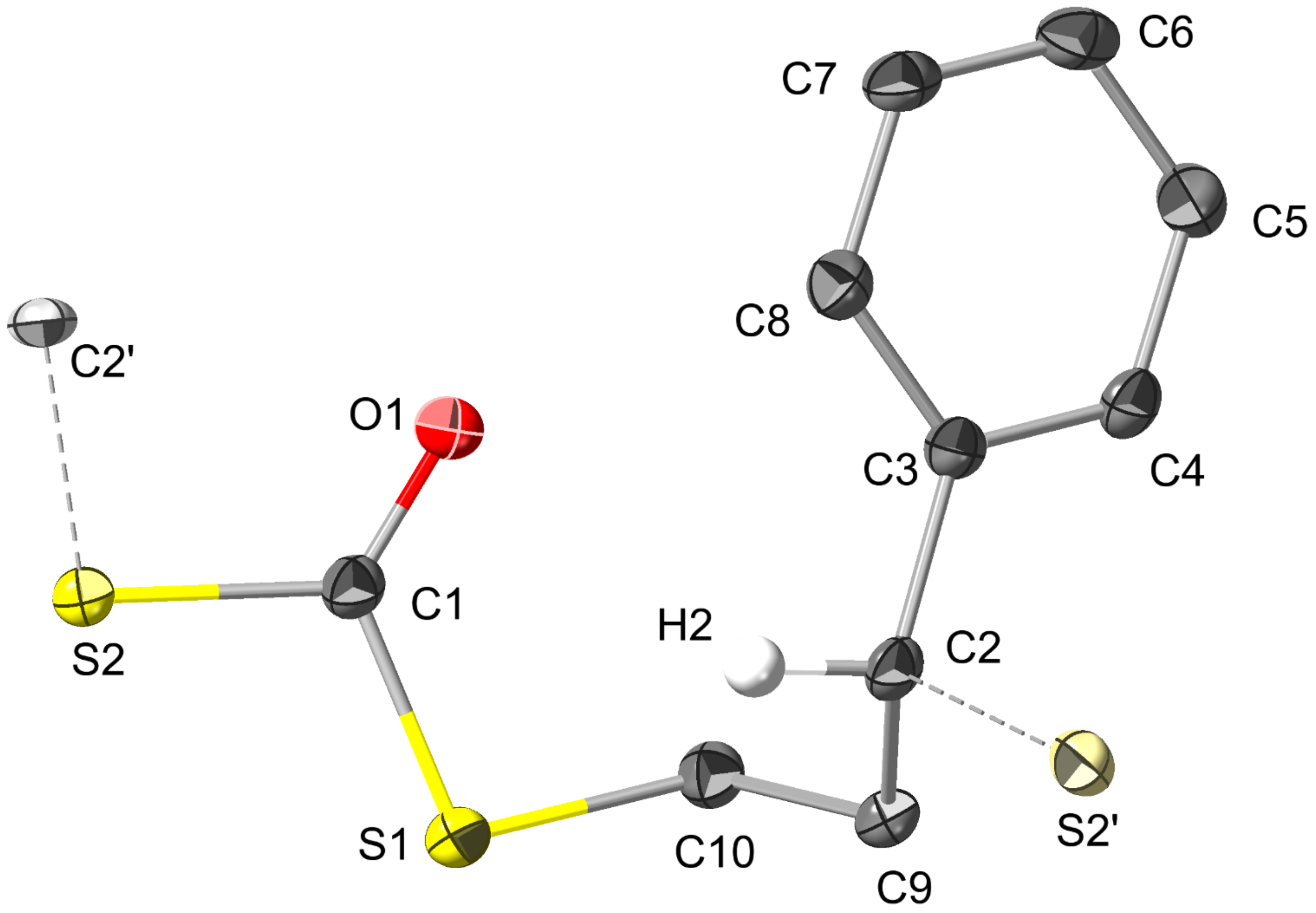

Figure S5. Structure showing asymmetric unit of **POTT** with ellipsoids with anisotropic displacement factors at 50% probability. Hydrogen atoms have been omitted for clarity. Selected interatomic distances (Å), bond angles (deg), and torsion angles (deg): S2–C2′ 1.844(3), S2–C1 1.782(3), C1–O1 1.203(4), S1–C1 1.765(3), S1–C10 1.825(3), C10–C9 1.528(4), C9–C2 1.522(4), C2–C3 1.514(4), C2′–S2–C1 98.88(14), S2–C1–O1 123.5(2), S2–C1–S1 111.34(16), O1–C1–S1 125.2(2), C1–S1–C10 100.41(13), S1–C10–C9 112.6(2), C10–C9–C2 113.7(2), C9–C2–C3 116.2(3), C2′–S2–C1–O1 –10.6(3), C2′–S2–C1–S1 169.84(15), S2–C1–S1–C10 168.58(16), O1–C1–S1–C10 –11.0(3), C1–S1–C10–C9 110.364(5),[11] S1–C10–C9–C2 –70.5(3), C10–C9–C2–C3 –69.3(3), C9–C2–C3–C4 –49.1(4).

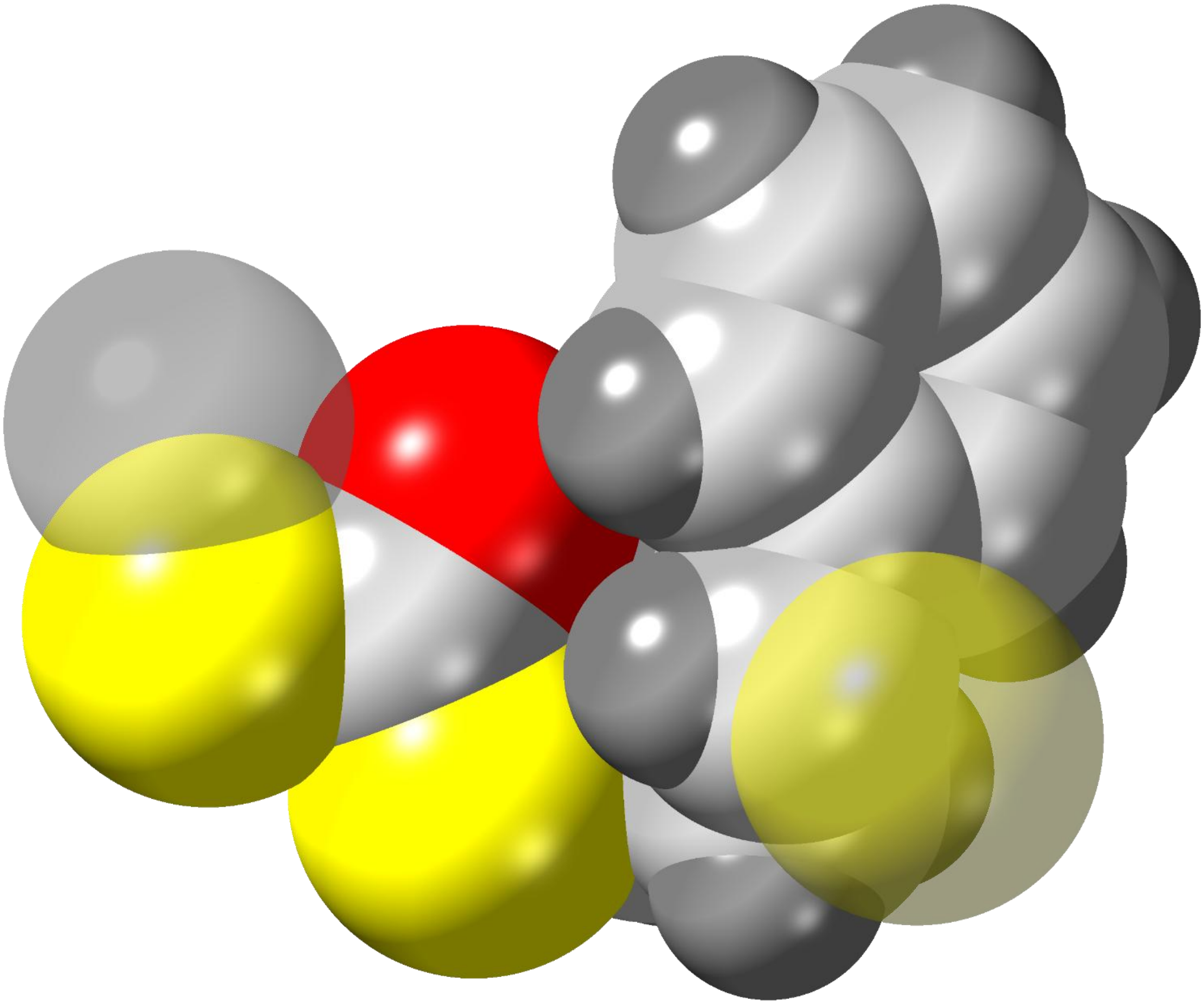

Figure S6. Space filling representation of the molecular structure of **POTT**. Repeat unit carbon and sulfur appear transparent.

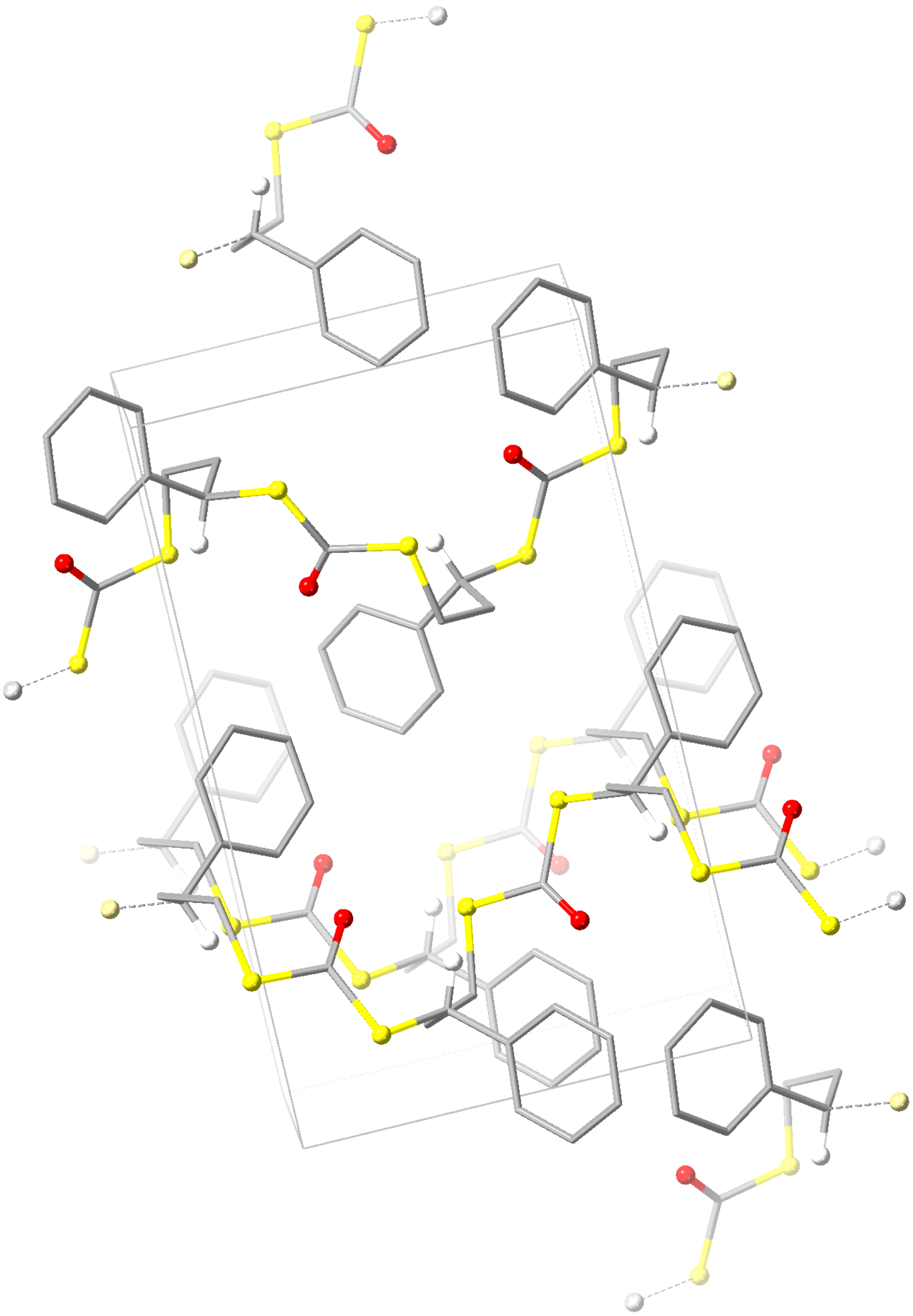

Figure S7. Crystal packing diagram of the unit cell of **POTT**. Hydrogen atoms have been omitted for clarity. Dash lines indicate repeat unit.

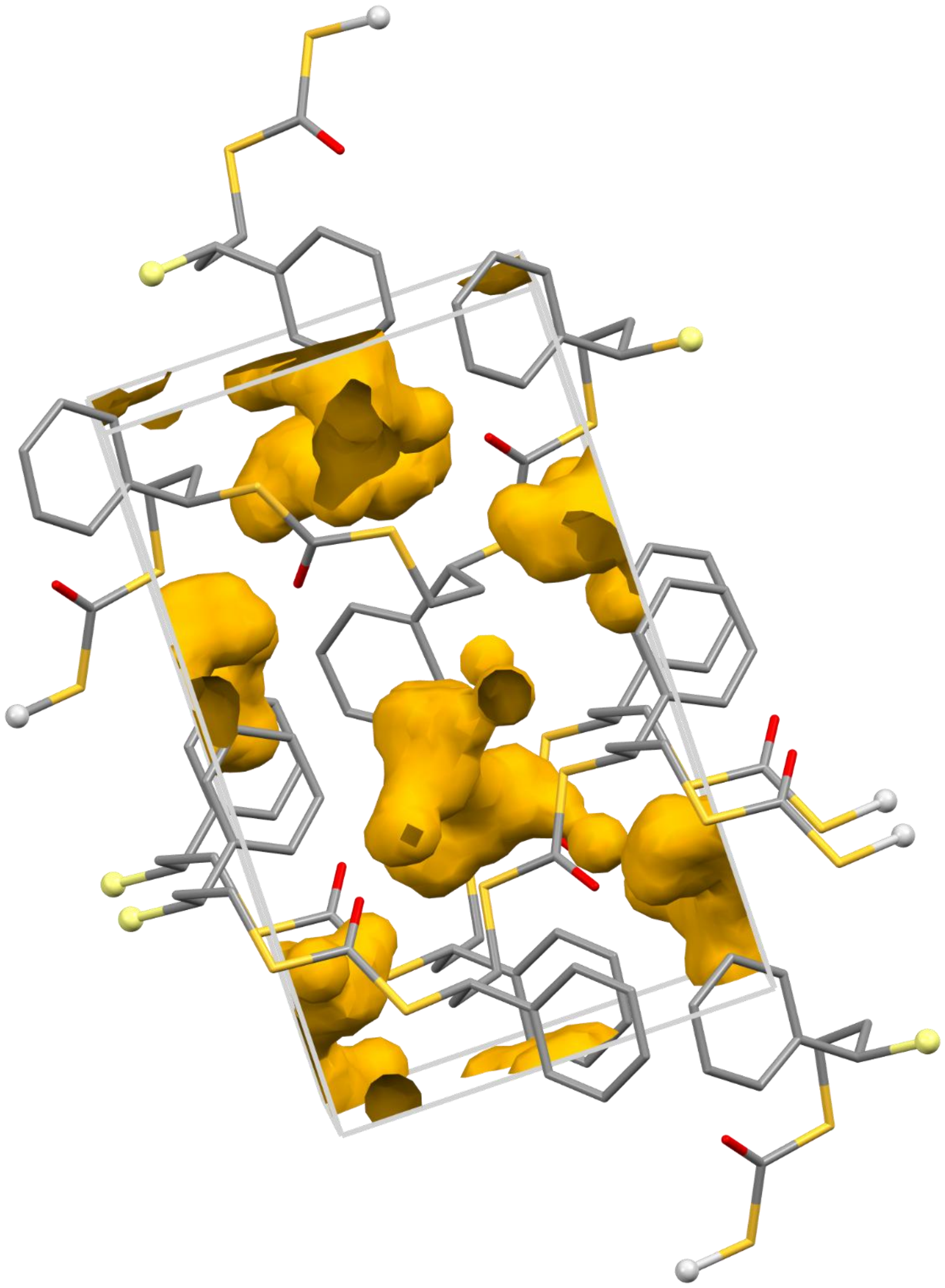

Figure S8. Crystal packing diagram of the unit cell of **POTT** showing void spaces (spherical probe radius: 0.6 Å). Hydrogen atoms have been omitted for clarity. Light gray and light-yellow ball and stick model atoms indicate repeat unit.

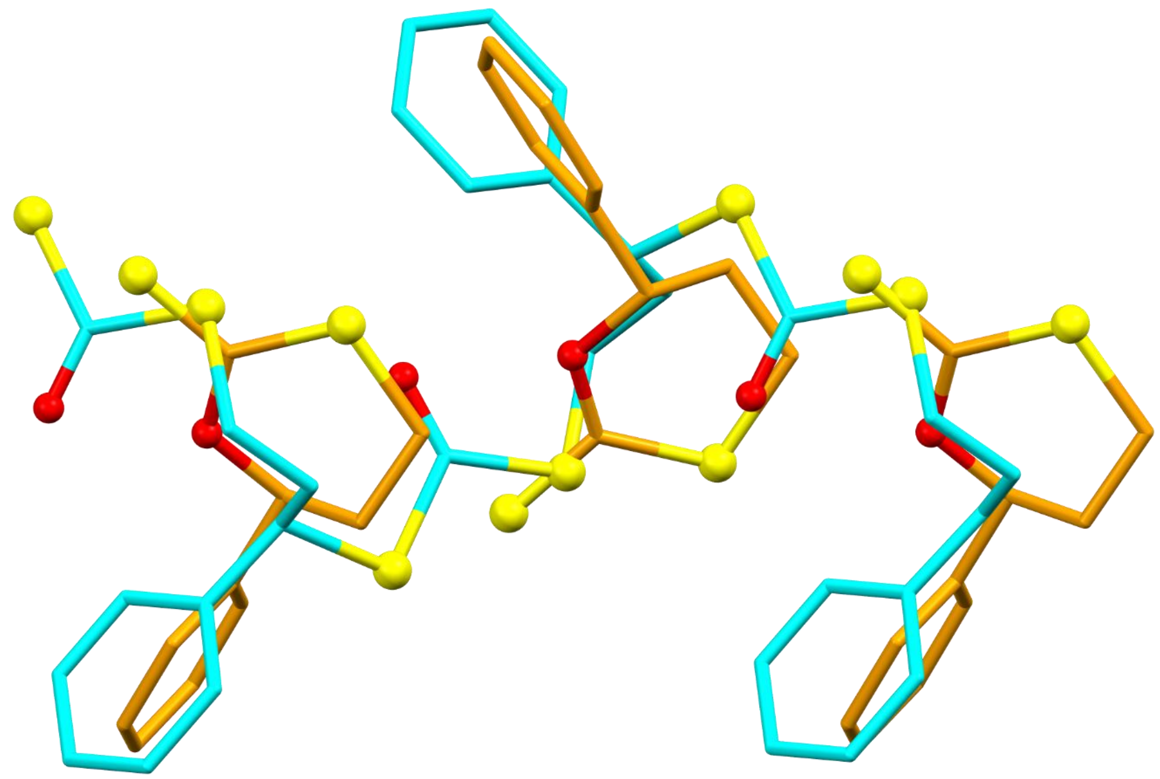

Figure S9. Three-unit overlay representation of the molecular XRD structures of **OTT** (orange) and **POTT** (light blue). Hydrogen atoms have been omitted for clarity.

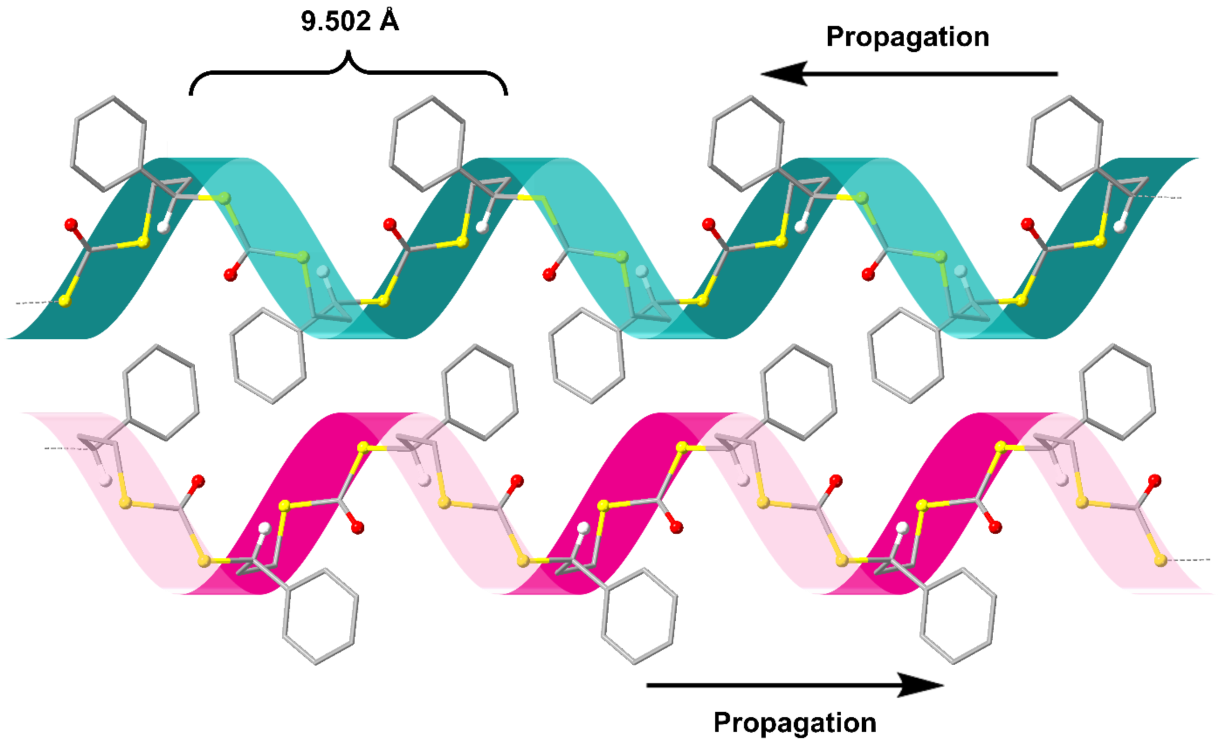

Figure S10. Helical orientation of **POTT** and propagation in antiparallel direction.

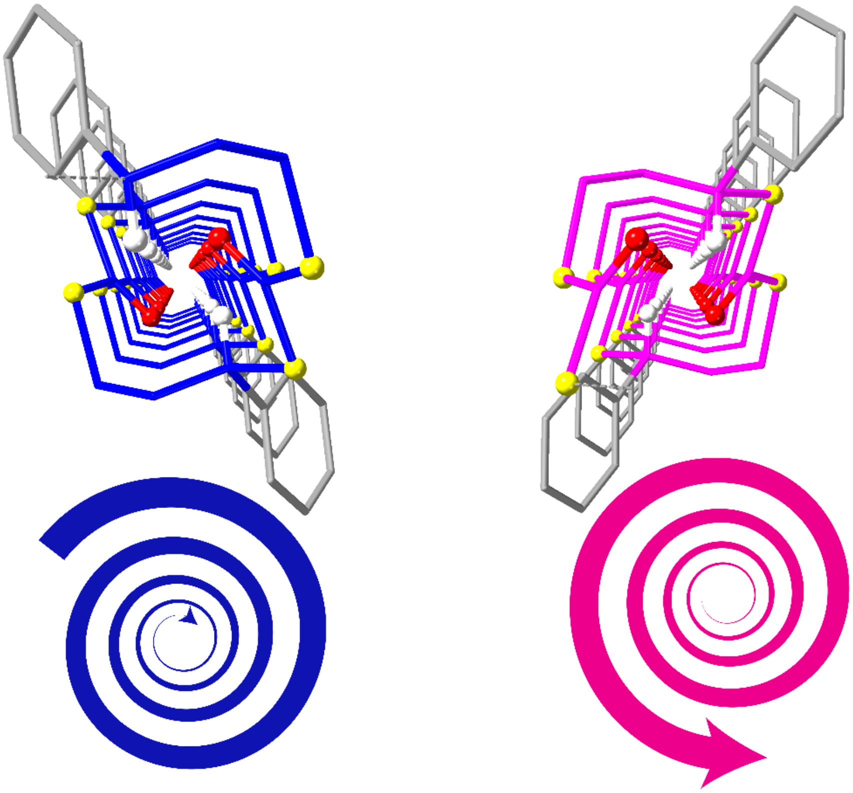

Figure S11. Internal view of helical **POTT** and propagation in antiparallel direction.

Table S1. Crystal data and refinement details for **OTT** and **POTT**.

	**OTT**	**POTT**
Empirical formula	$C_{10}H_{10}OS_2$	$C_{10}H_{10}OS_2$
M_r	210.30	210.30
Crystal size [mm]	0.338 × 0.268 × 0.164	0.216 × 0.168 × 0.13
Crystal system	orthorhombic	orthorhombic
Space group	$P2_12_12_1$	$P2_12_12_1$
a [Å], α [°]	7.7152(5), 90	6.9020(8), 90
b [Å], β [°]	9.7927(7), 90	9.5011(12), 90
c [Å], γ [°]	12.8333(7), 90	15.4822(19), 90
V [Å^3]	969.59(11)	1015.3(2)
Z	4	4
$\rho_{calcd.}$ [g cm^{-3}]	1.441	1.376
F(000)	440	440
μ [mm^{-1}]	0.502	0.480
T_{max}/T_{min}	0.862 / 0.820	0.862 / 0.810
hkl range	±11, ±14, –16 +18	±8, ±12, ±19
2θ range [°]	10.464 – 126.02	10.06 – 108.408
Measured refl.	54449	54535
Unique refl. [R_{int}]	3233 [0.0562]	2245 [0.0631]
Data / restr. / param.	3233 / 0 / 118	2245 / 0 / 118
Goodness-of-fit	1.077	1.172
$R1$ (I>2σ(I))	0.0205	0.0312
$wR2$ (I>2σ(I))	0.0507	0.0687
$R1$ (all data)	0.0222	0.0342
$wR2$ (all data)	0.0513	0.0702
Flack parameter	–0.008(16)	0.01(3)
Resid. electron dens. [e Å^{-3}]	0.28 / –0.18	0.34 / –0.28

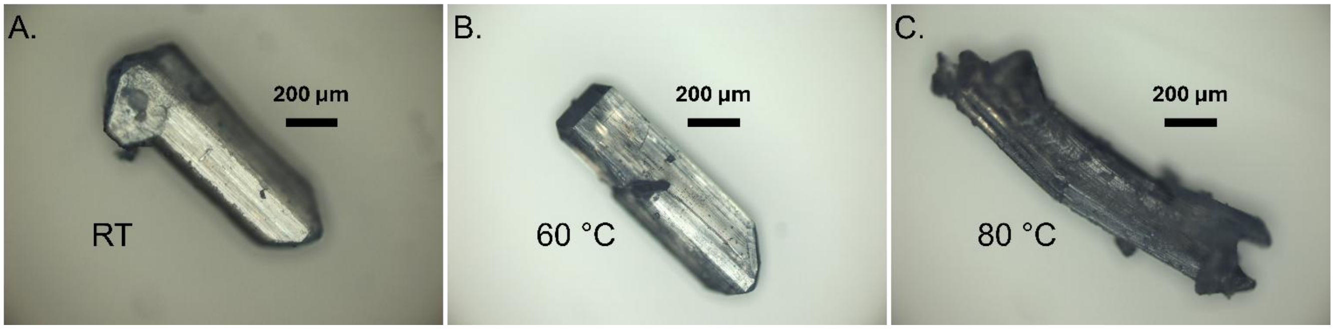

Figure S12. Polymerized crystals of **POTT** at RT (A), 60 (B), and 80 °C (C).

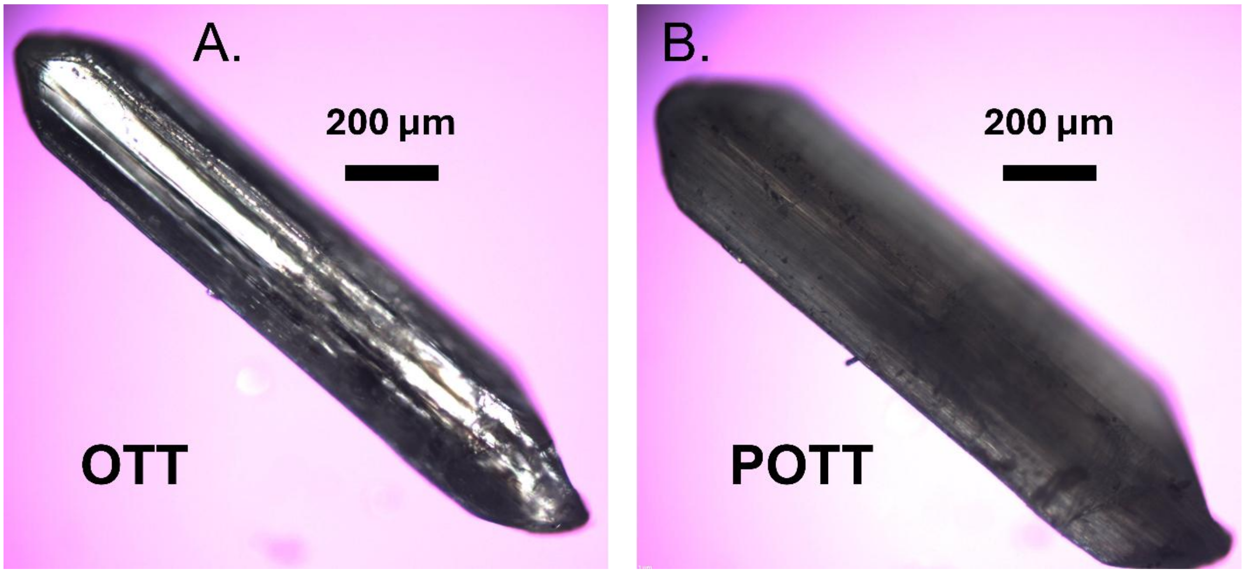

Figure S13. Crystals of **OTT** and **POTT** under polarized light before (A) and after (B) polymerization.

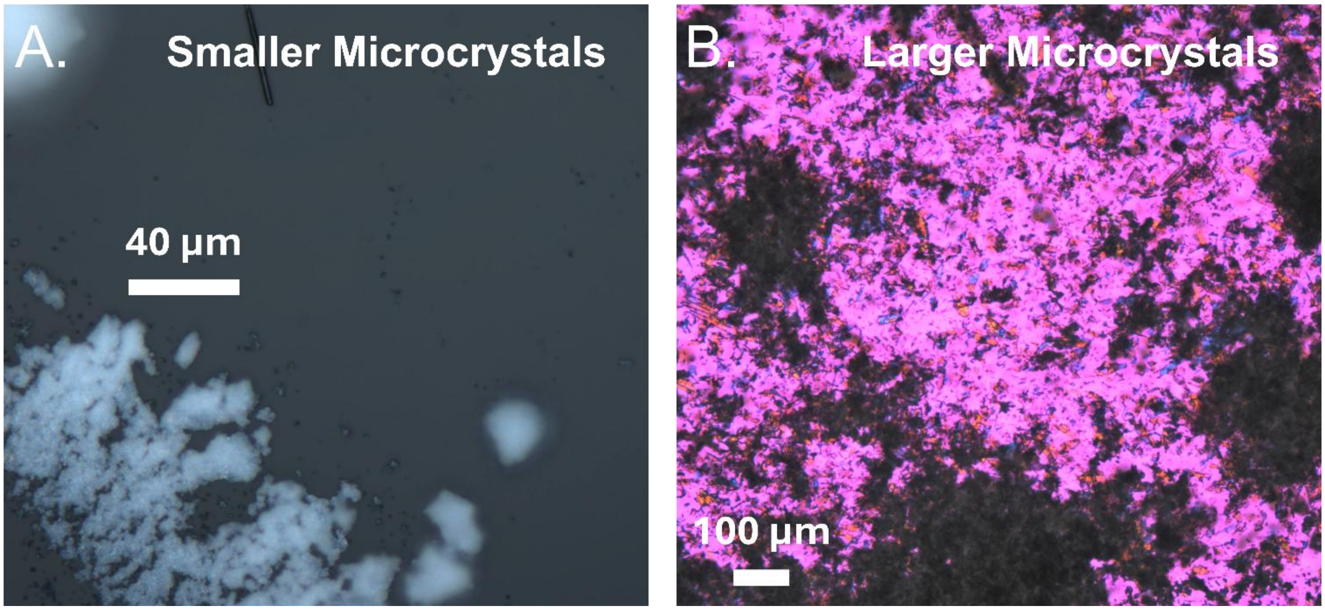

Figure S14. Microcrystalline samples of **OTT** under white light (A) and filter polarized light (B).

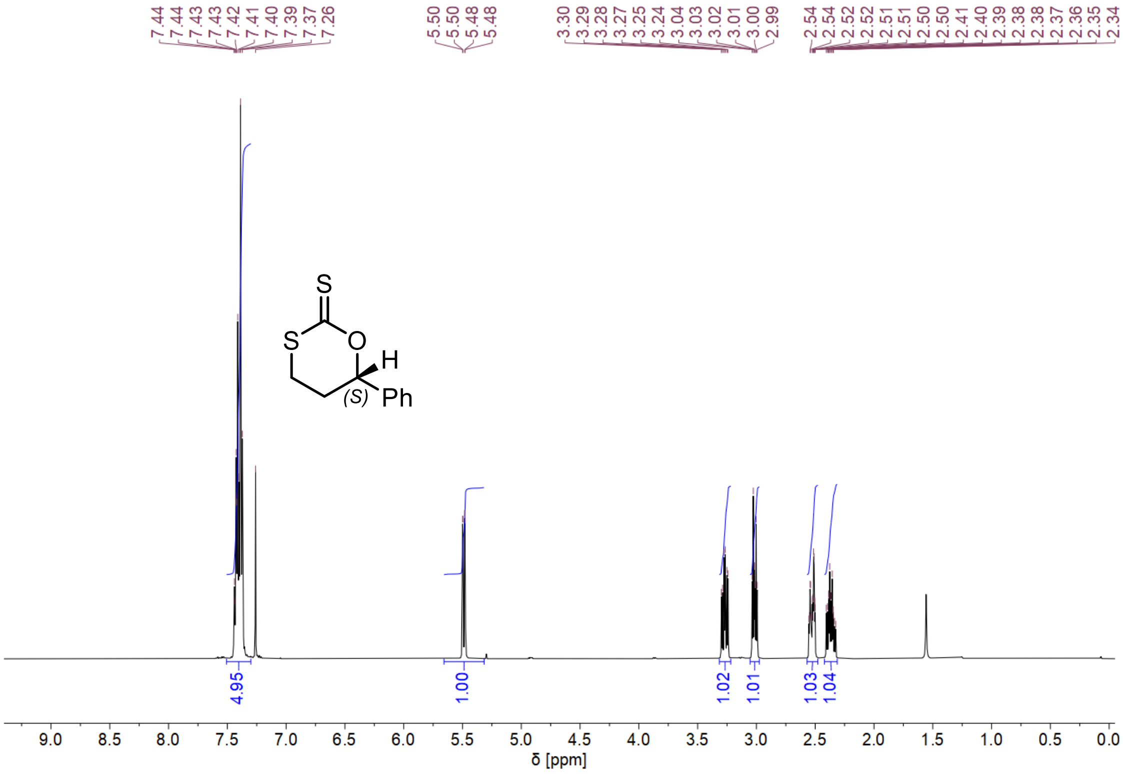

Figure S15. ^{1}H NMR spectrum of **OTT** ($CDCl_3$, 500.1 MHz).

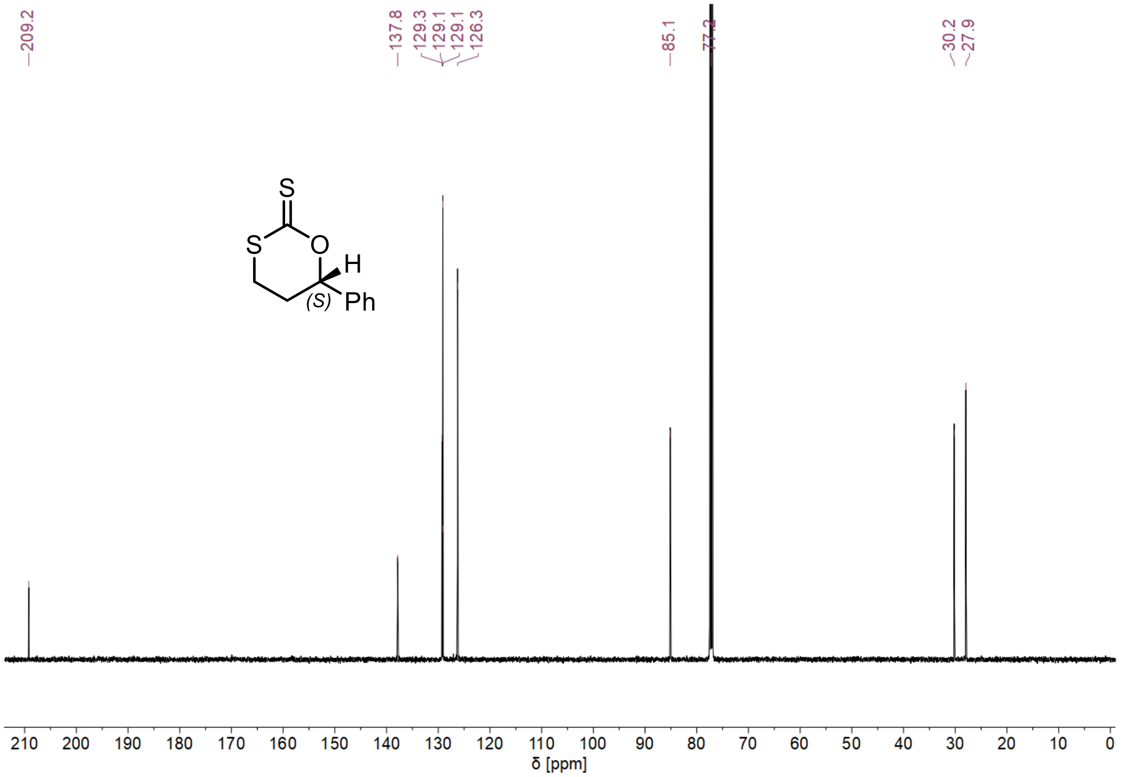

Figure S16. ^{13}C NMR spectrum of **OTT** ($CDCl_3$, 125.8 MHz).

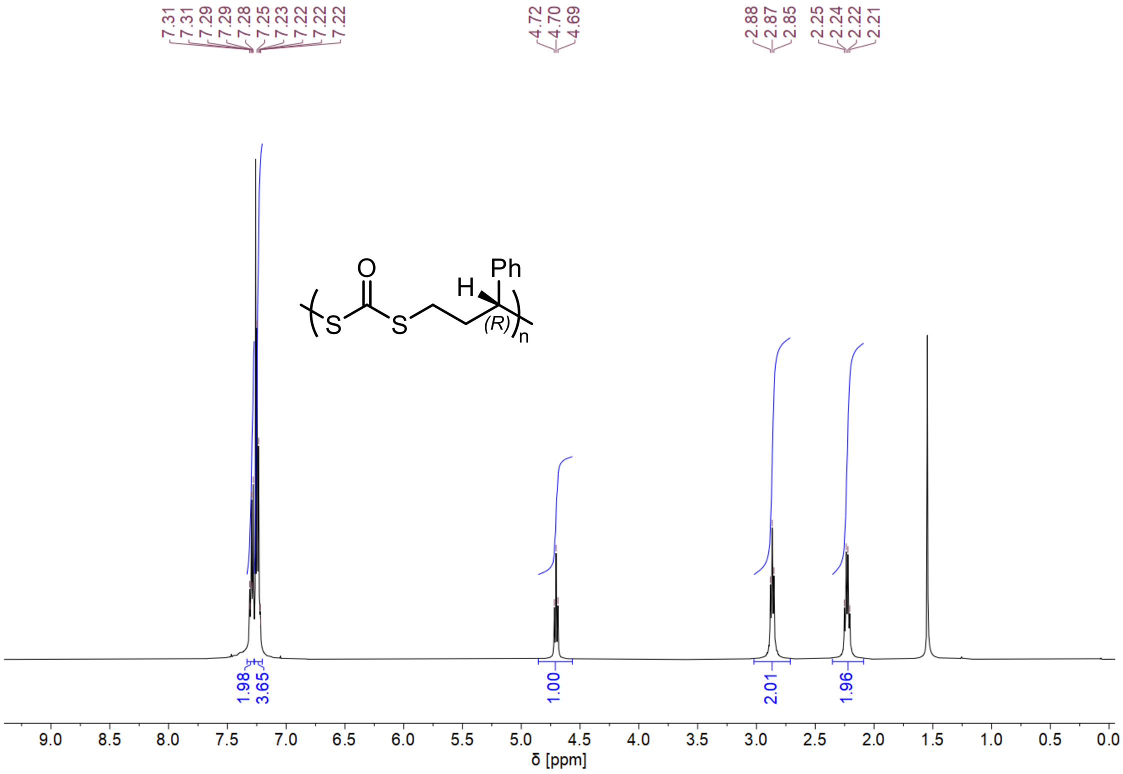

Figure S17. ^{1}H NMR spectrum of **POTT** ($CDCl_3$, 500.1 MHz).

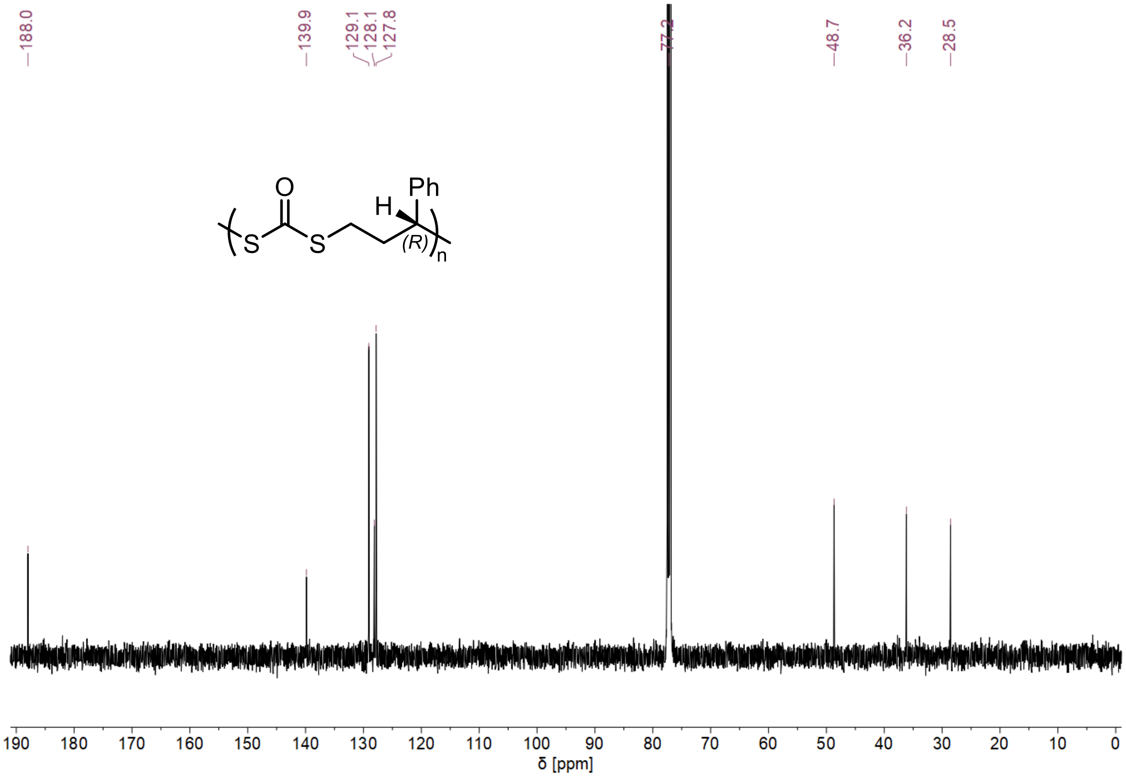

Figure S18. ^{13}C NMR spectrum of **POTT** ($CDCl_3$, 125.8 MHz).

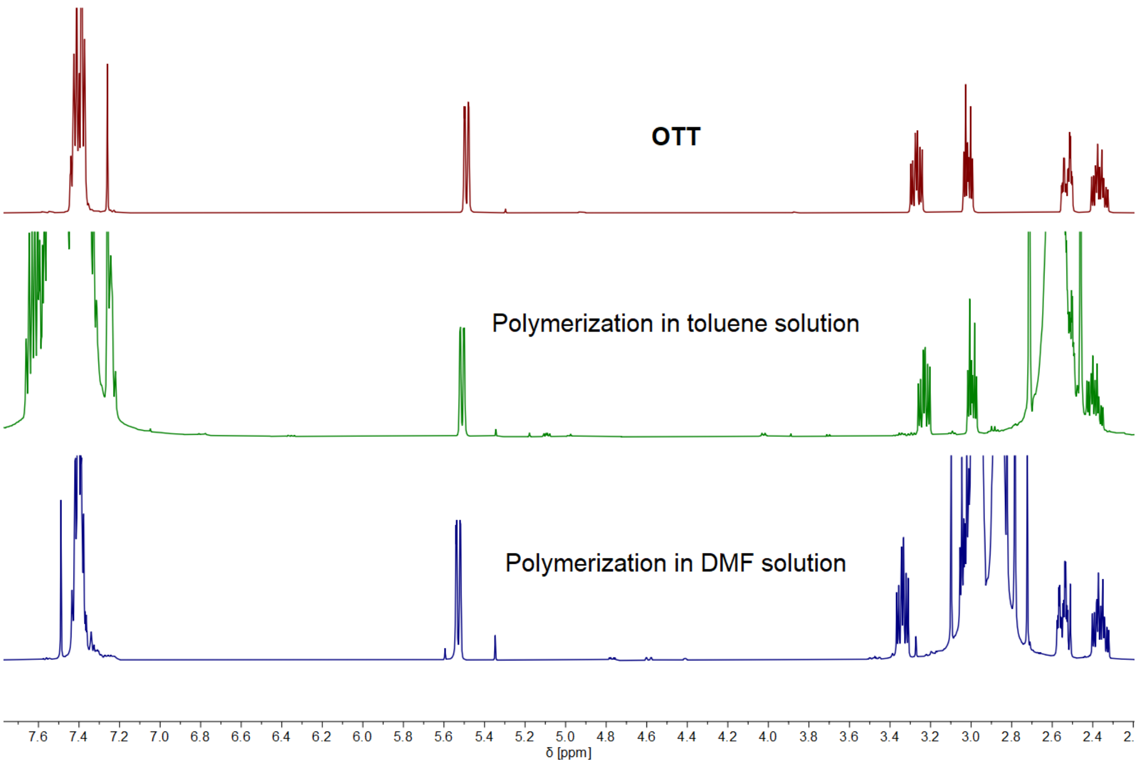

Figure S19. ^{1}H NMR spectrum comparison of **OTT** polymerization attempts in solutions of toluene and DMF ($CDCl_3$, 500.1 MHz).

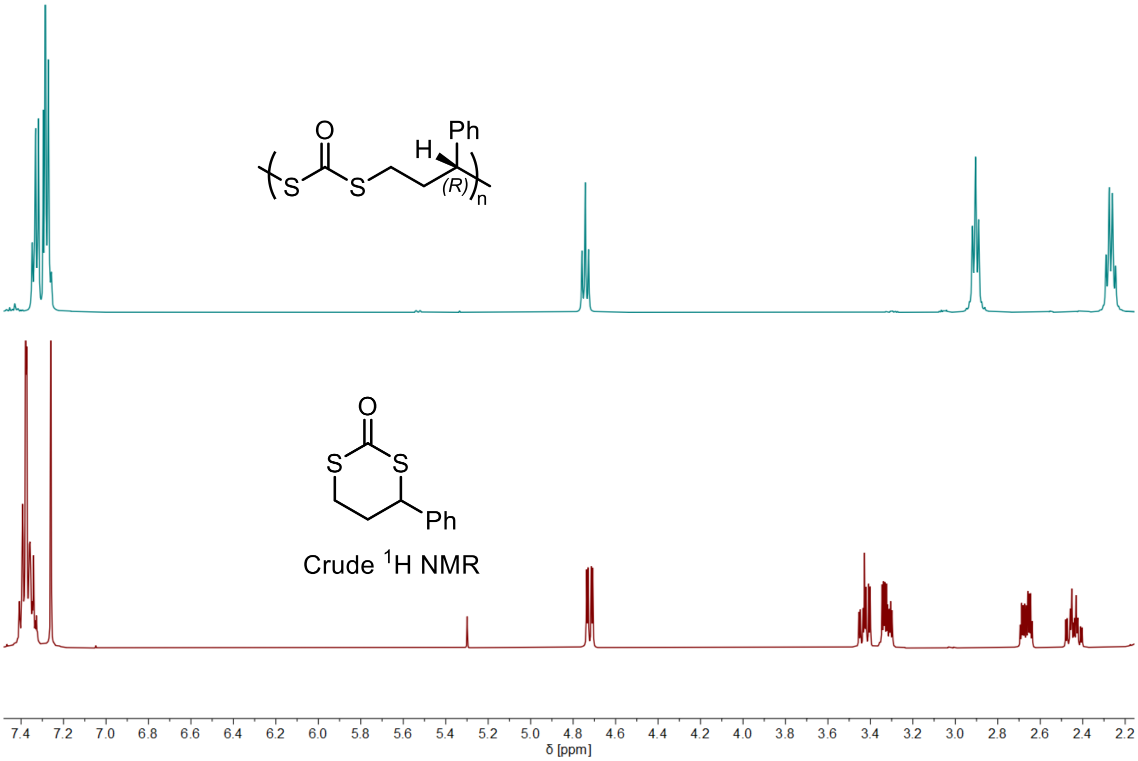

Figure S20. ^{1}H NMR spectrum comparison of depolymerized **POTT** ($CDCl_3$, 500.1 MHz).

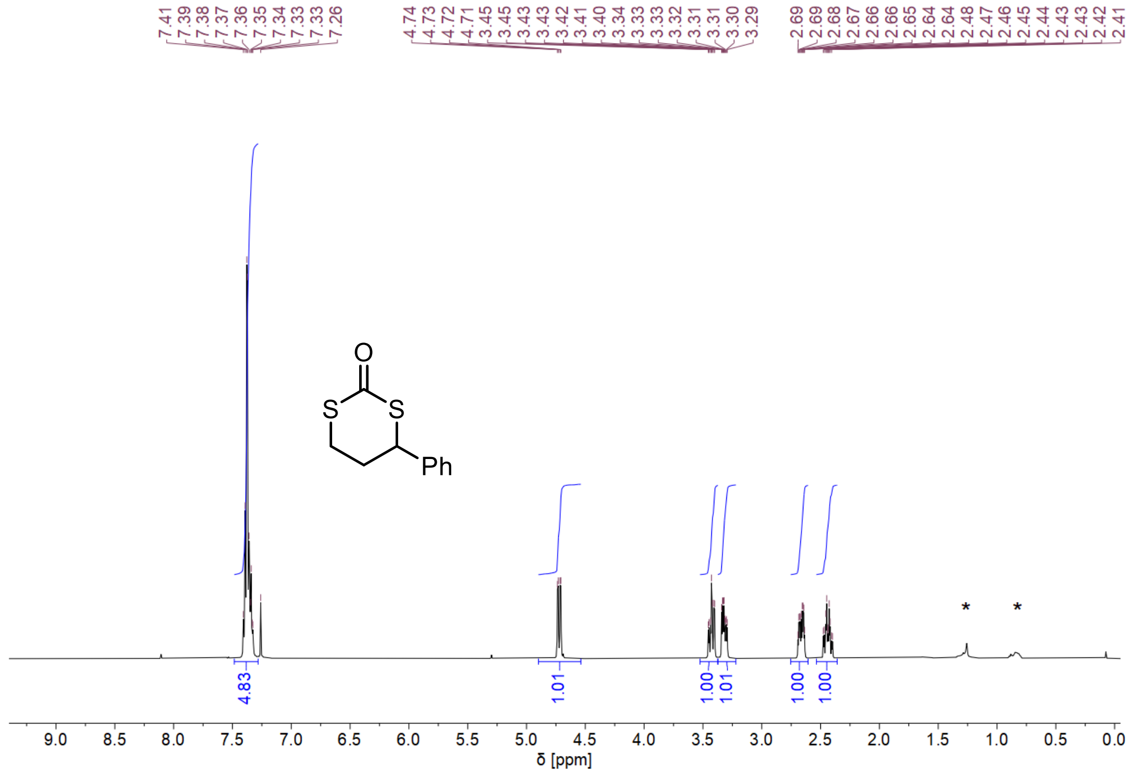

Figure S21. ^{1}H NMR spectrum of depolymerized **POTT** ($CDCl_3$, 500.1 MHz) *) indicates residual hexanes.

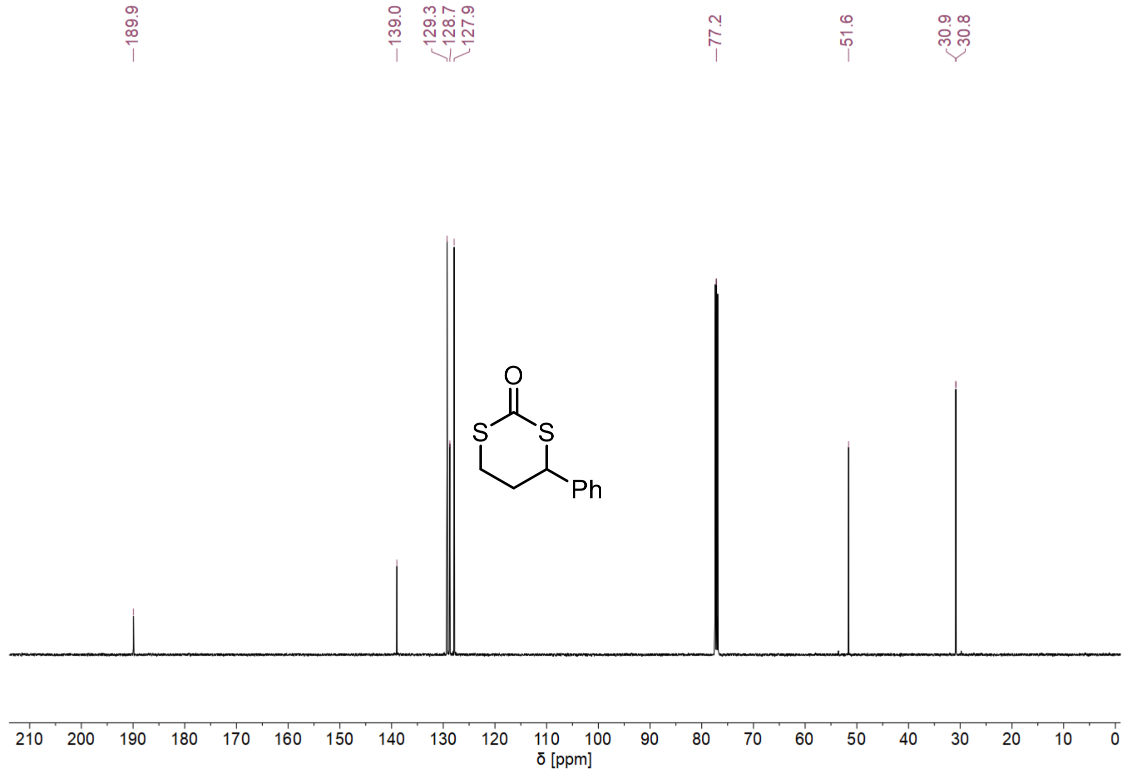

Figure S22. ^{13}C NMR spectrum of depolymerized **POTT** ($CDCl_3$, 125.8 MHz).

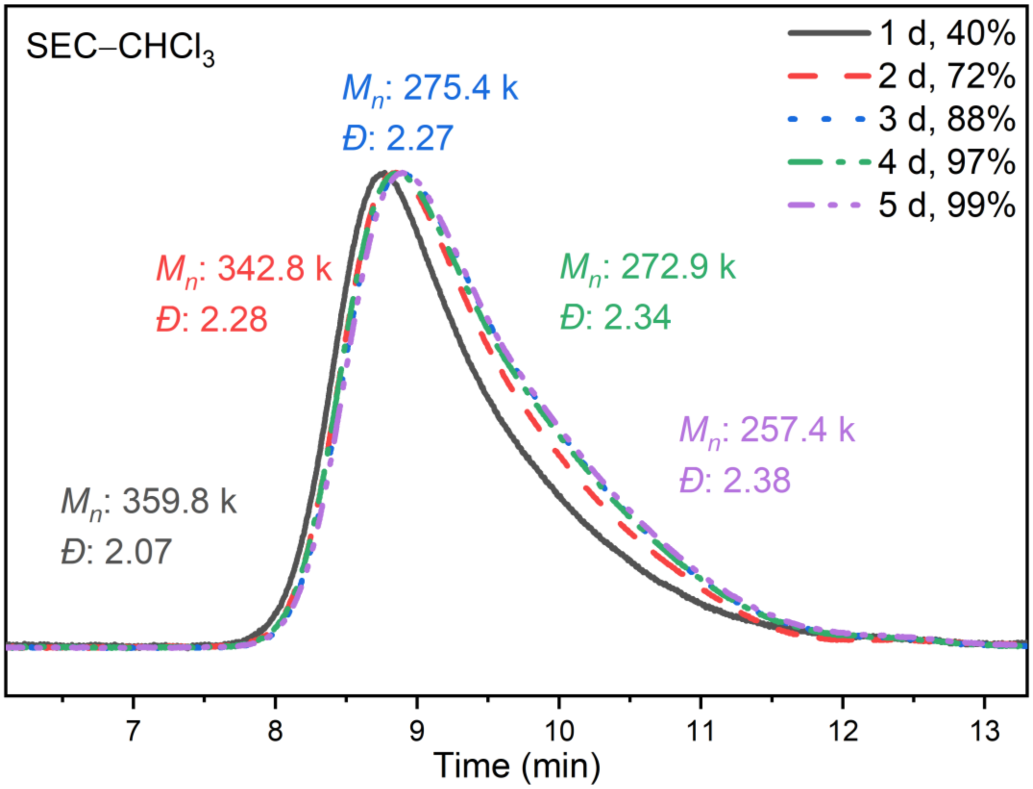

Figure S23. SEC trace of topochemical polymerization at room temperature.

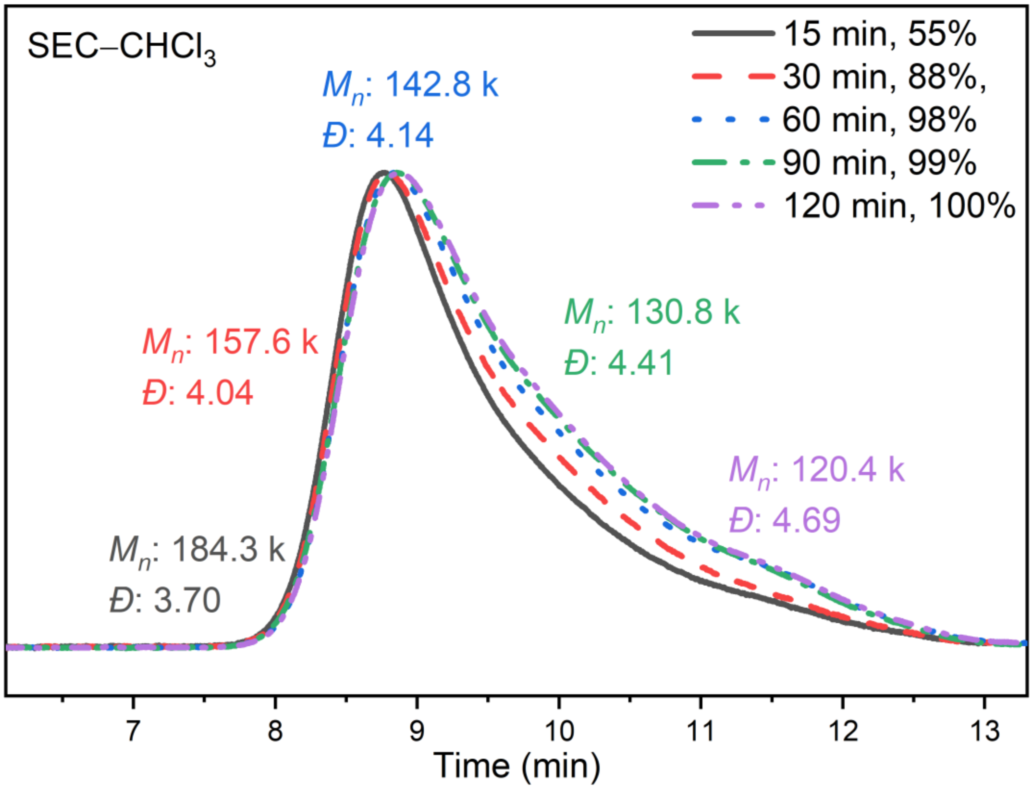

Figure S24. SEC trace of topochemical polymerization at 60 °C.

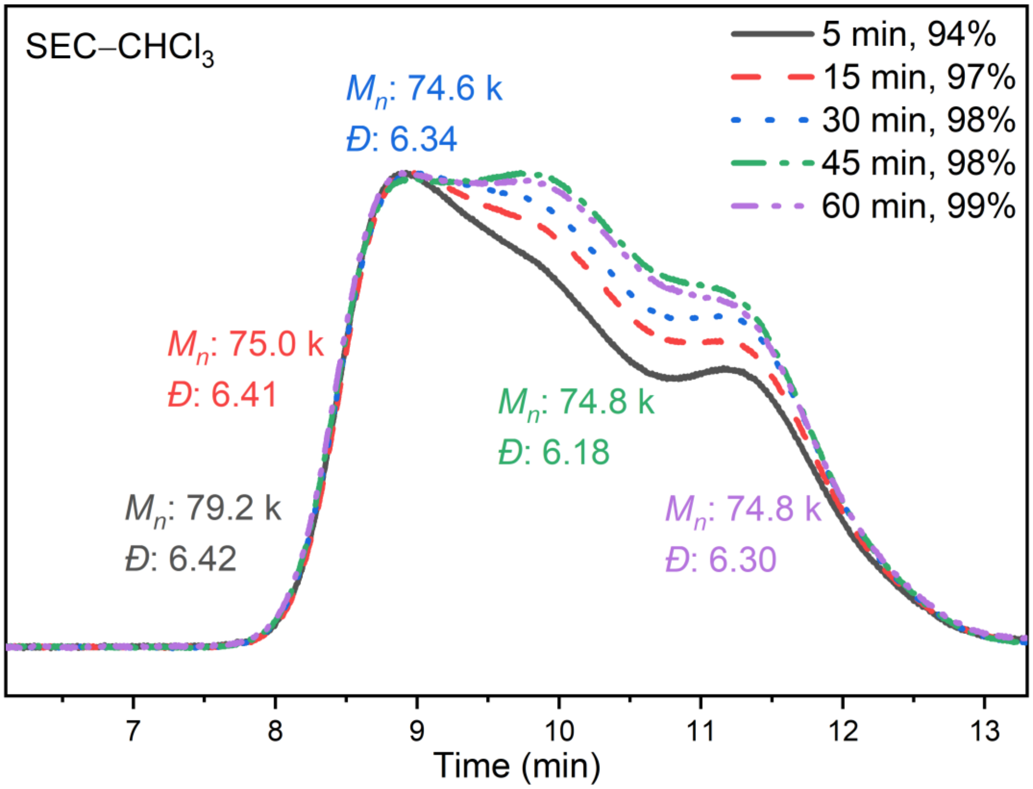

Figure S25. SEC trace of topochemical polymerization at 80 °C.

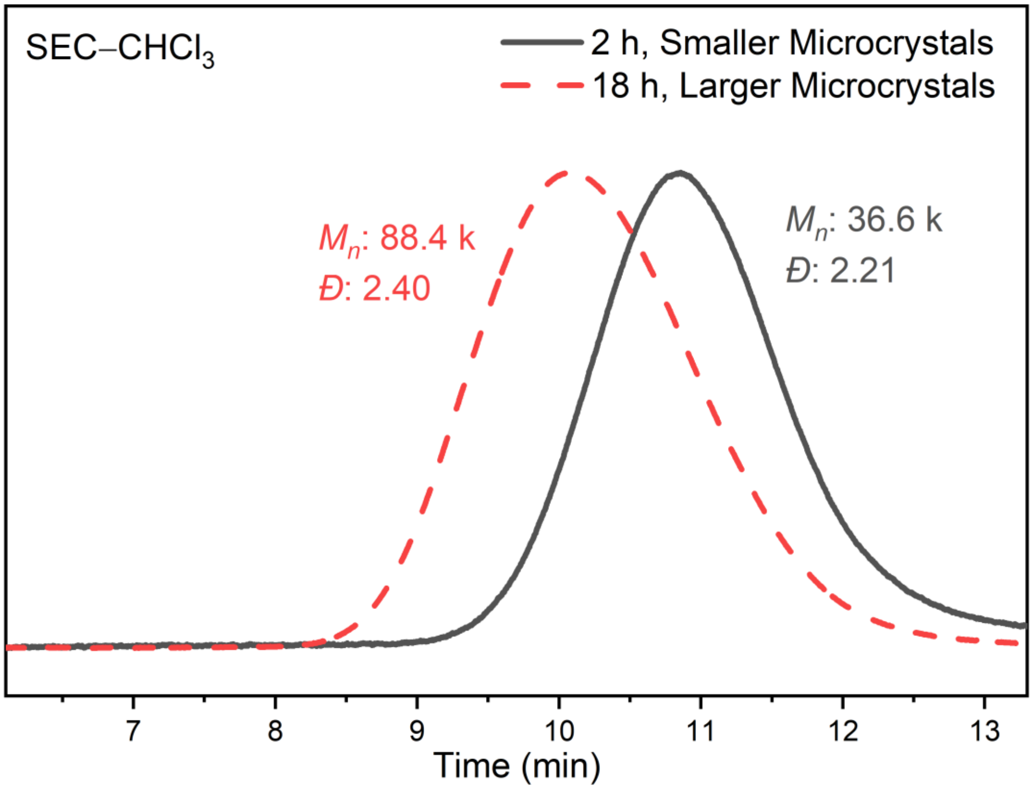

Figure S26. SEC trace of topochemical polymerization of microcrystalline OTT at 60 °C.

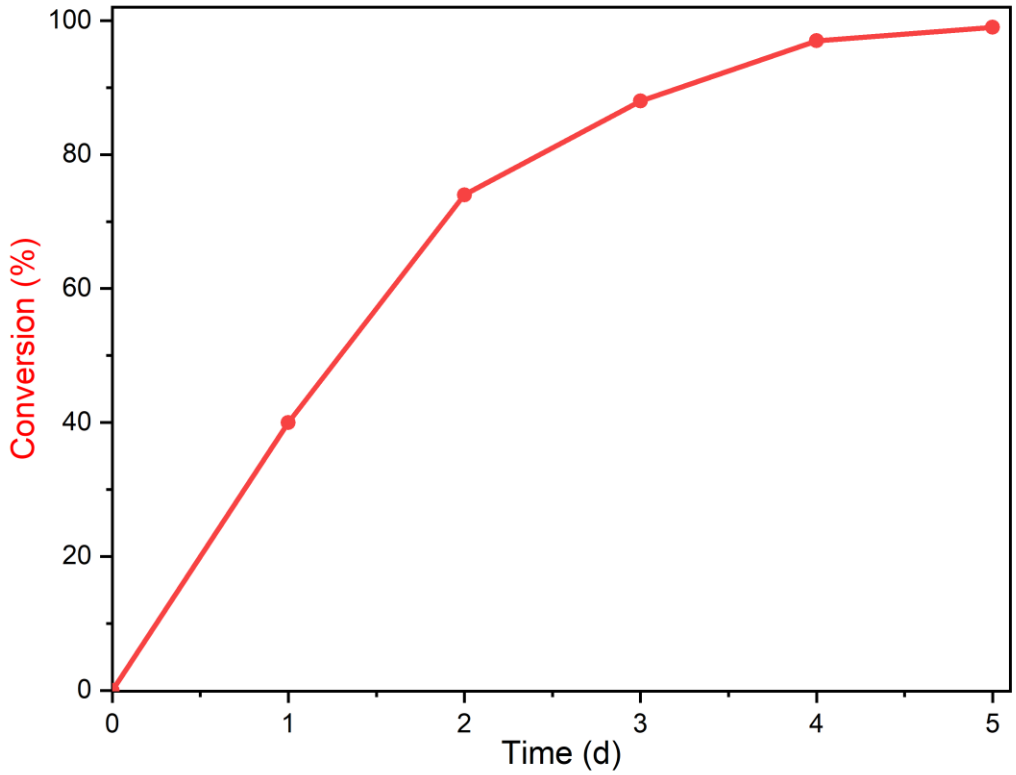

Figure S27. Conversion of topochemical polymerization at room temperature.

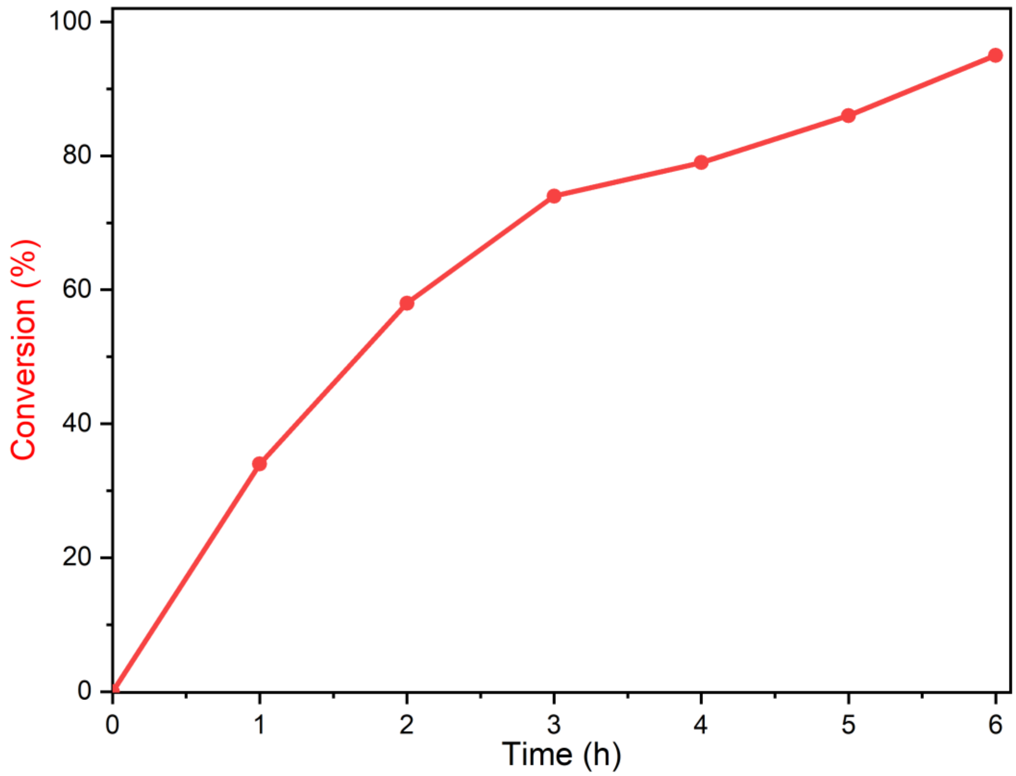

Figure S28. Conversion of topochemical polymerization at 40 °C.

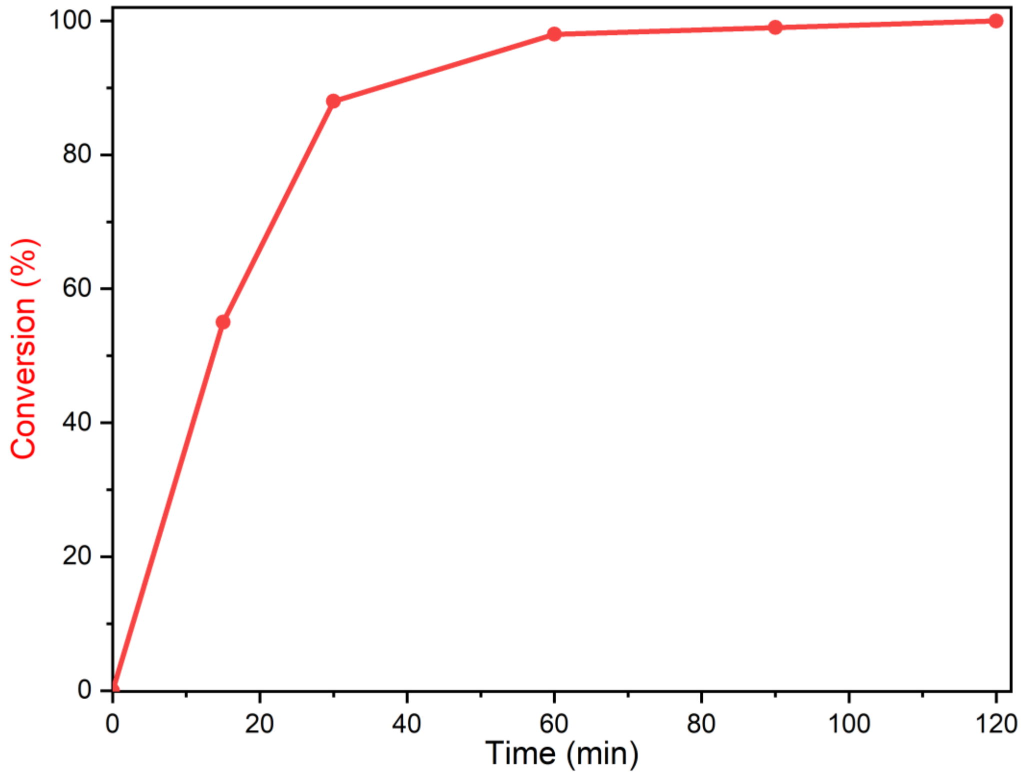

Figure S29. Conversion of topochemical polymerization at 60 °C.

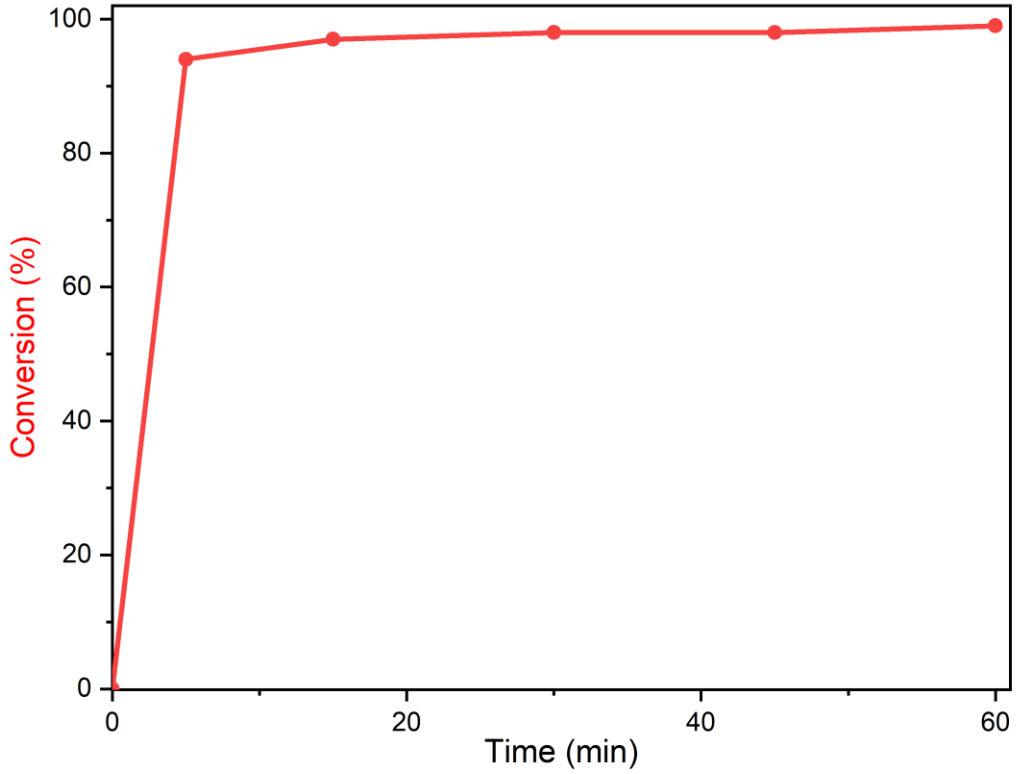

Figure S30. Conversion of topochemical polymerization at 80 °C.

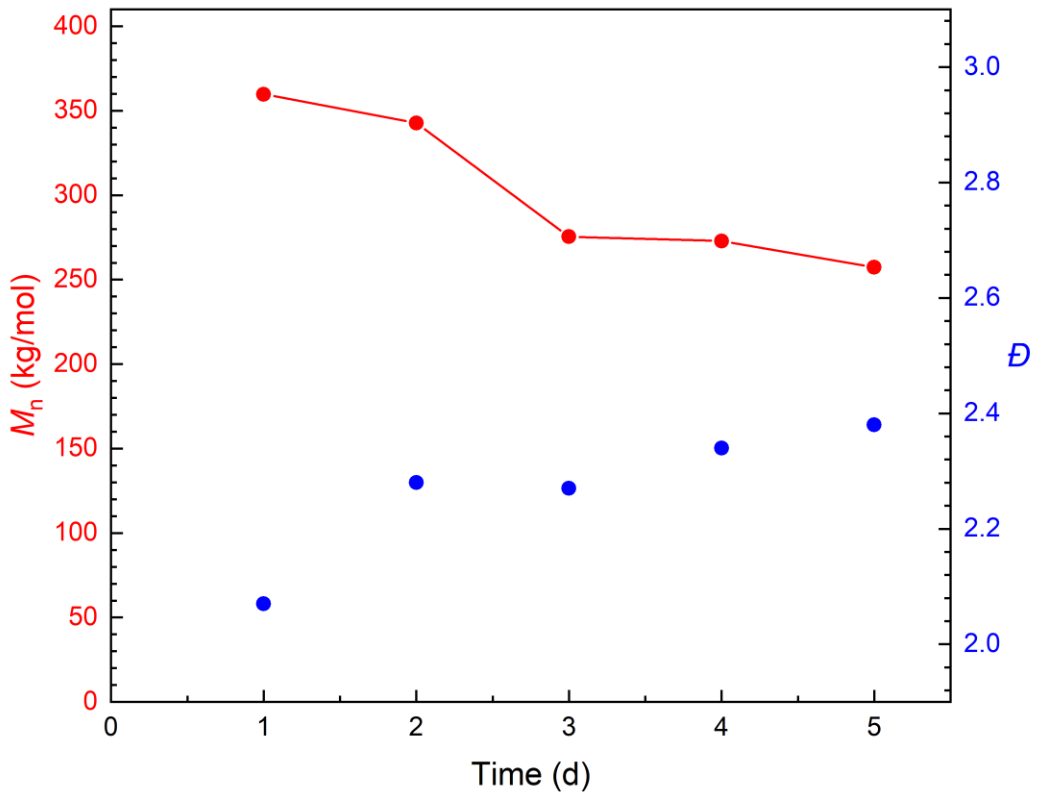

Figure S31. M_n and *Đ* of topochemical polymerization at room temperature.

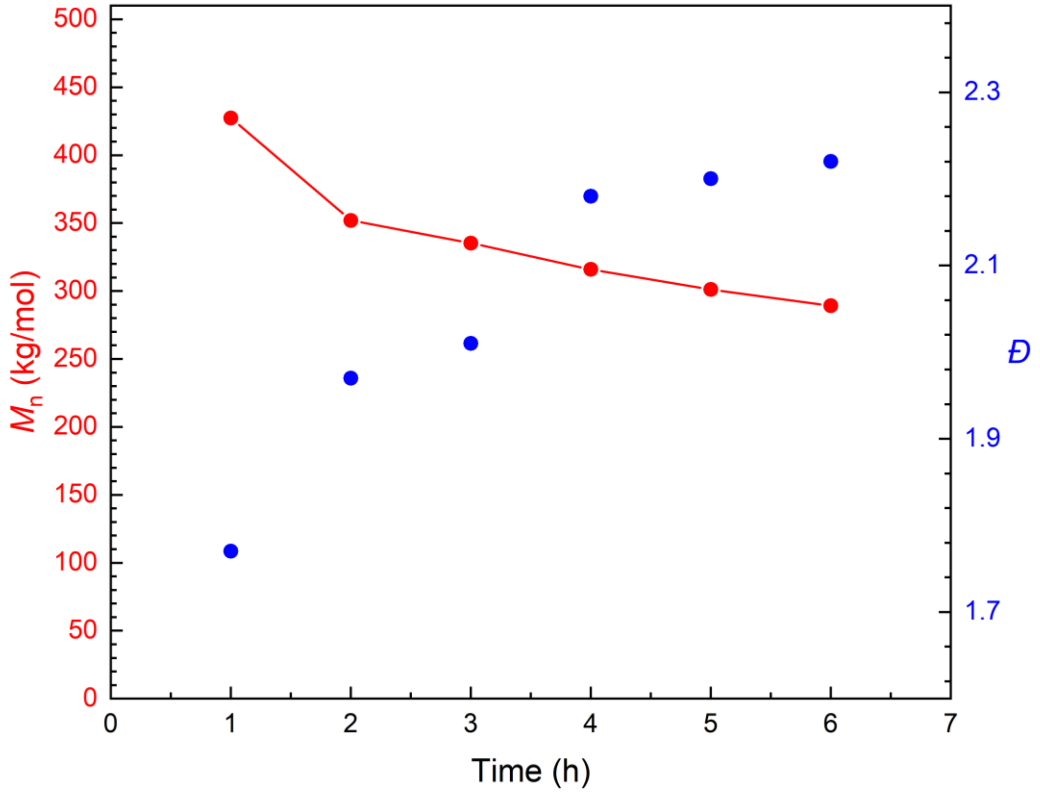

Figure S32. M_n and *Đ* of topochemical polymerization at 40 °C.

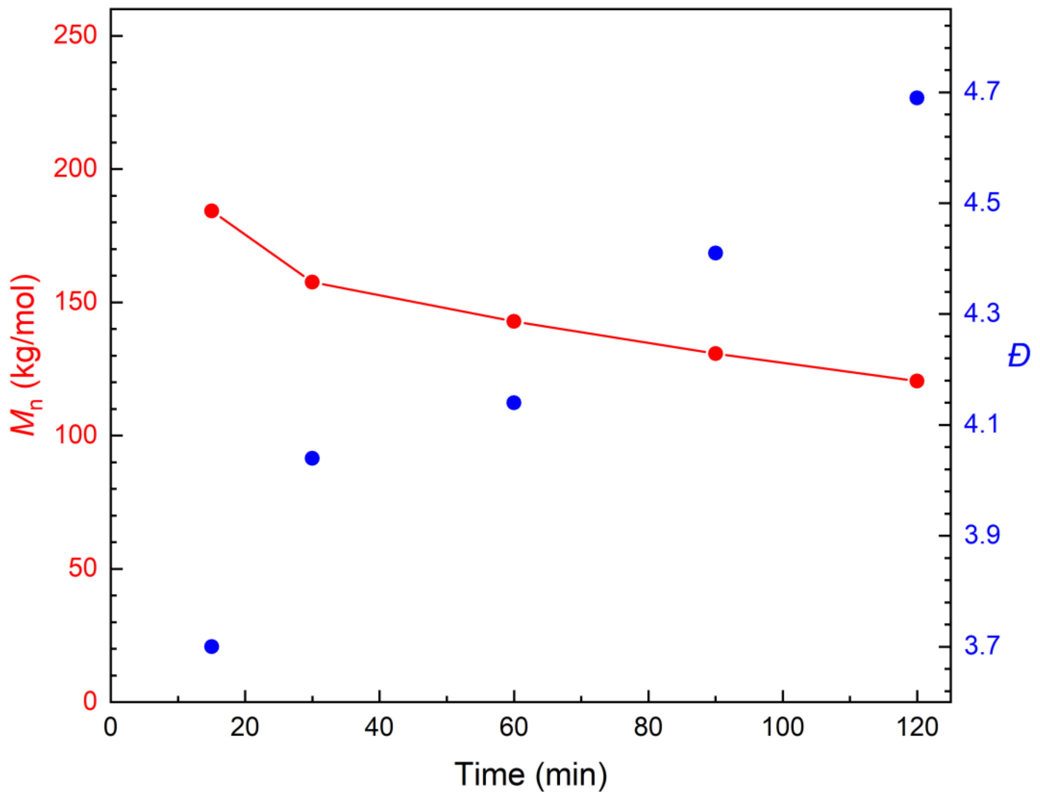

Figure S33. M_n and *Đ* of topochemical polymerization at 60 °C.

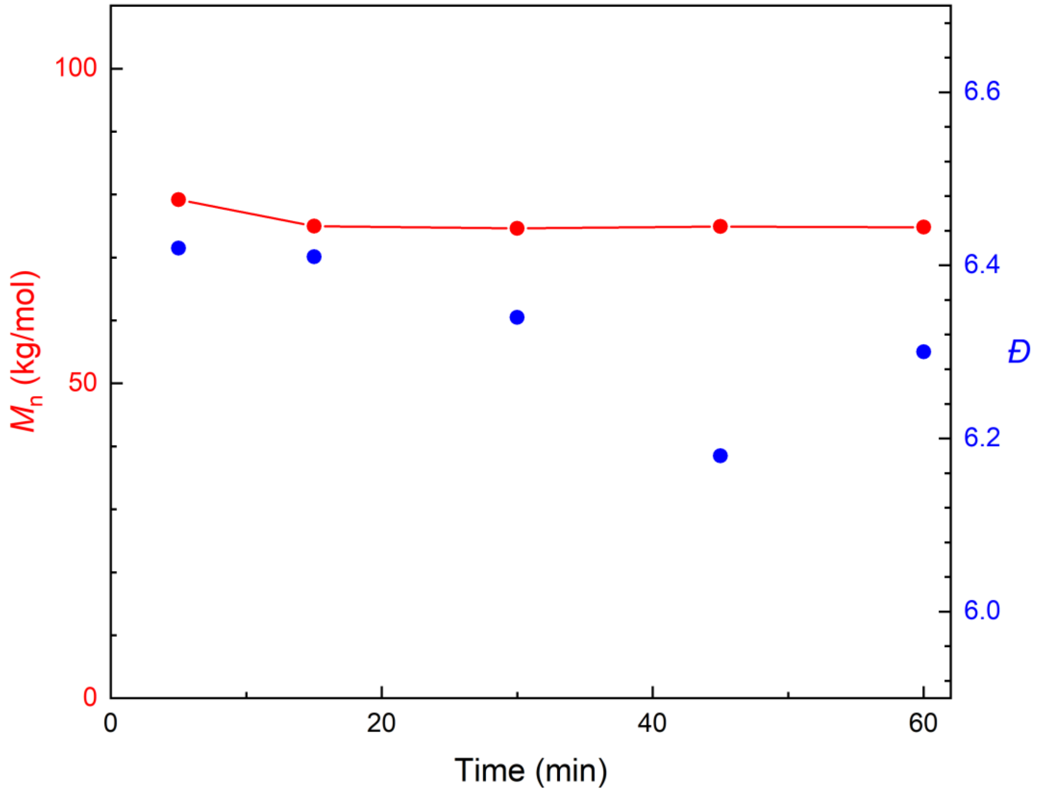

Figure S34. M_n and *Đ* of topochemical polymerization at 80 °C.

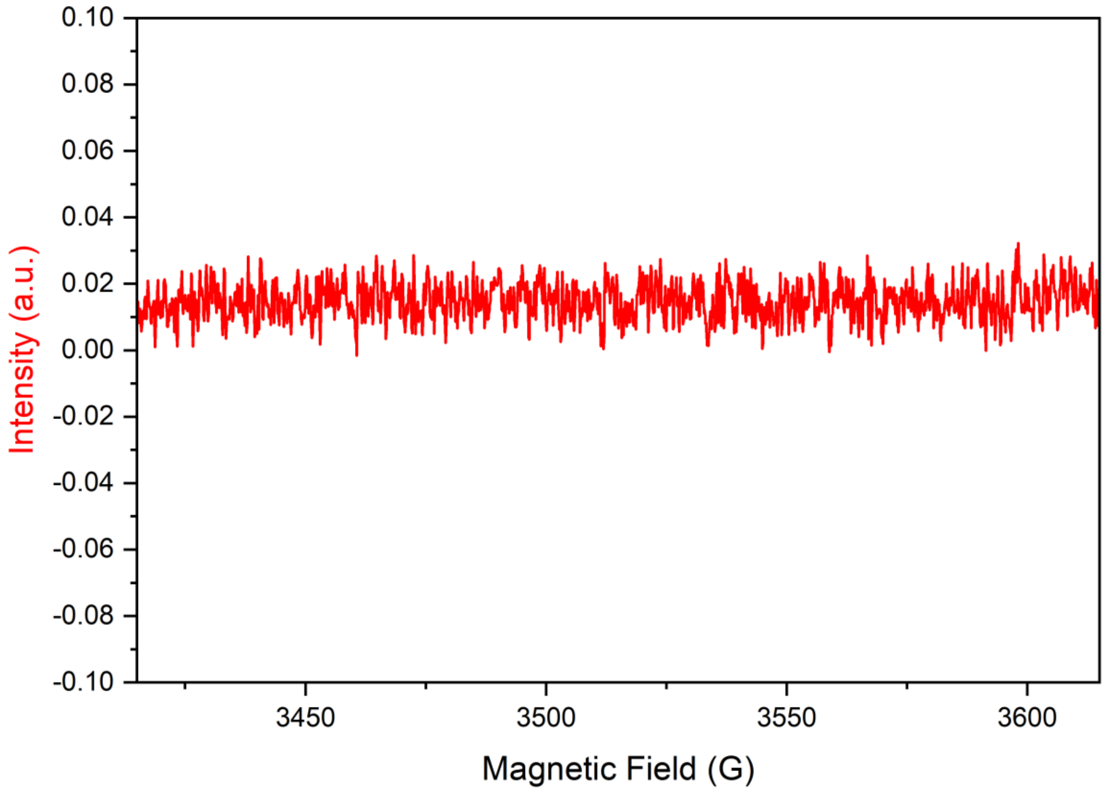

Figure S35. EPR spectrum of topochemical polymerization after 2 h at 40 °C with 200 field sweep.

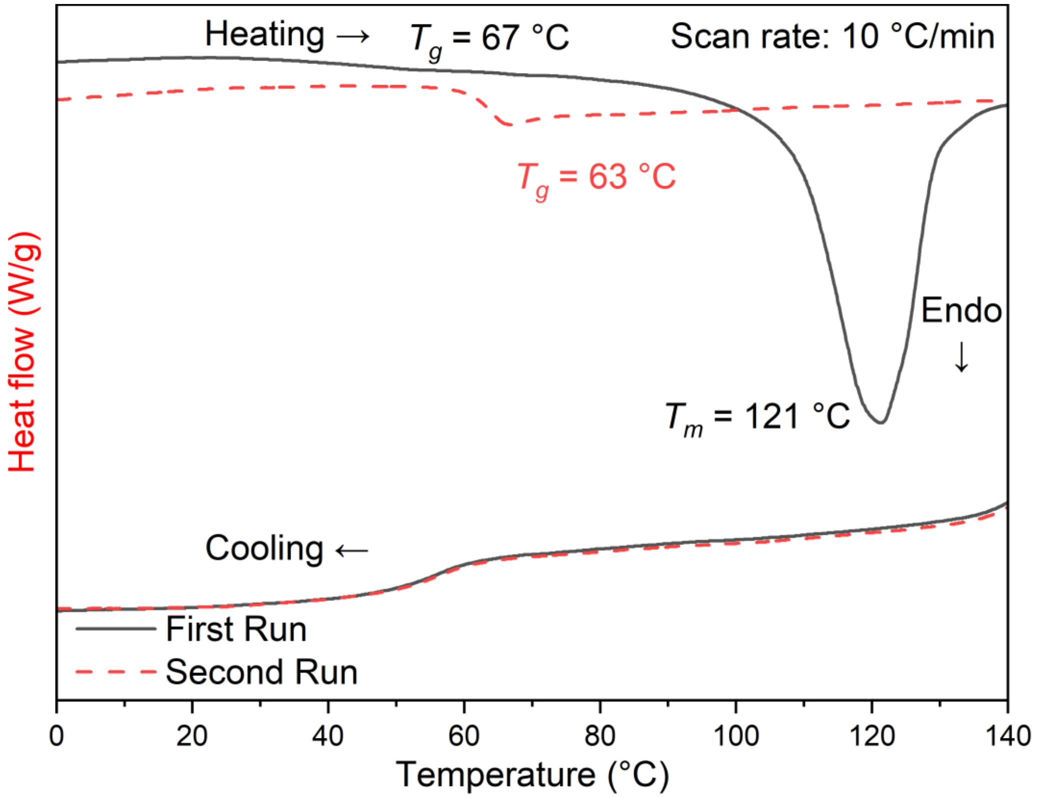

Figure S36. DSC of topochemical polymerization at room temperature.

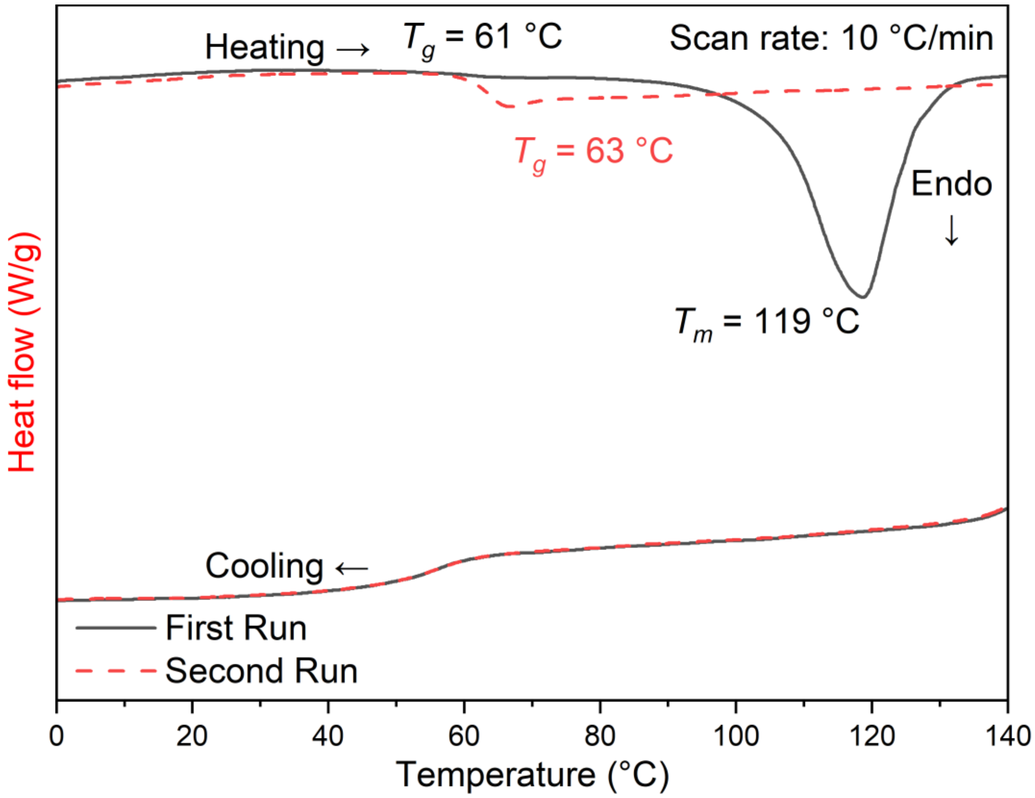

Figure S37. DSC of topochemical polymerization at 60 °C.

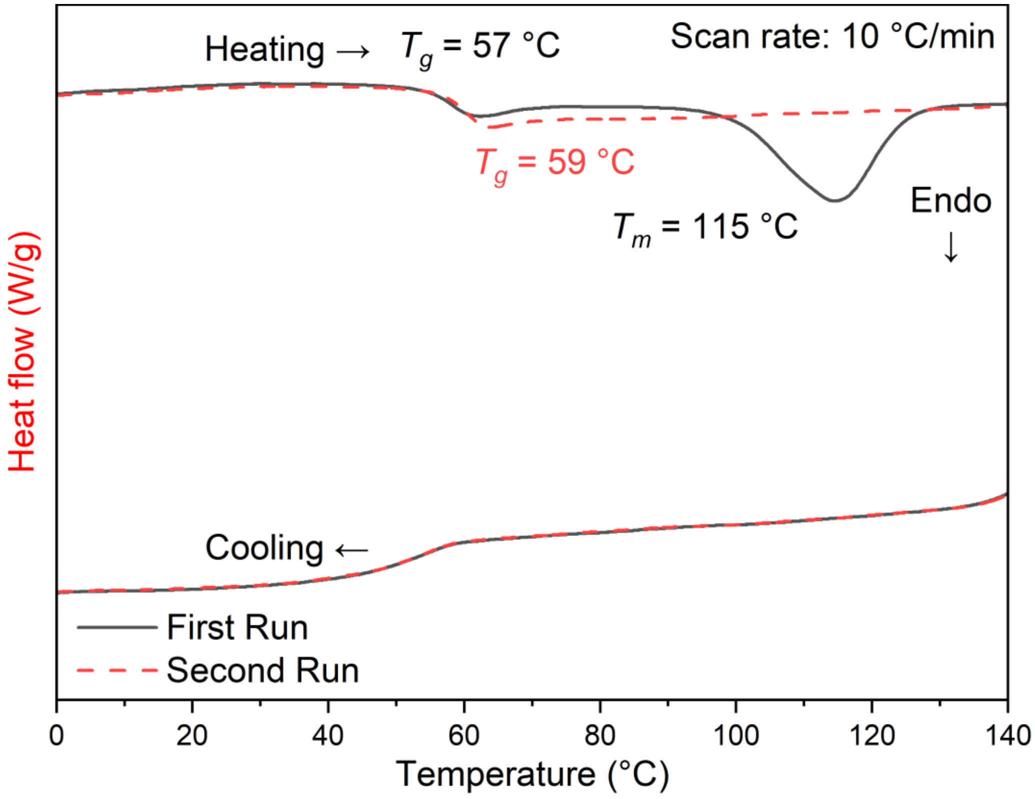

Figure S38. DSC of topochemical polymerization at room temperature 80 °C.

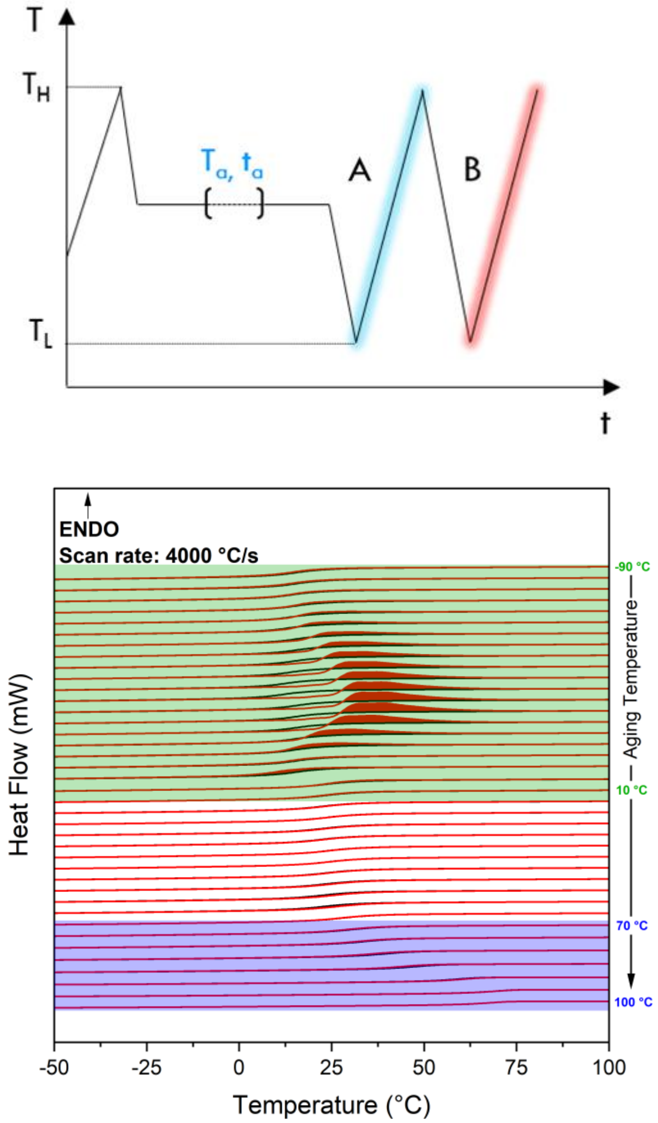

Figure S39. Top panel: Fast scanning calorimetry (FSC) temperature program followed for aging experiments on **OTT**. **Bottom panel:** Corresponding FSC data for aged (red thermograms) and reference unaged samples (black thermograms). Aging temperatures between –90 °C and 100 °C were used. They are indicated on the right of the graph. Enthalpic overshoots (red highlighted areas between the heating scans of the aged and unaged reference samples) are observed when the aging temperature is below the **OTT** glass transition temperature, T_g (green highlighted region). Data obtained for aging at 10 °C to 100 °C show a gradual shift of T_g, deduced from the step change in heat flow, to higher temperatures starting from aging temperatures of 70 °C (blue highlighted region), indicating the onset temperature for polymerization.

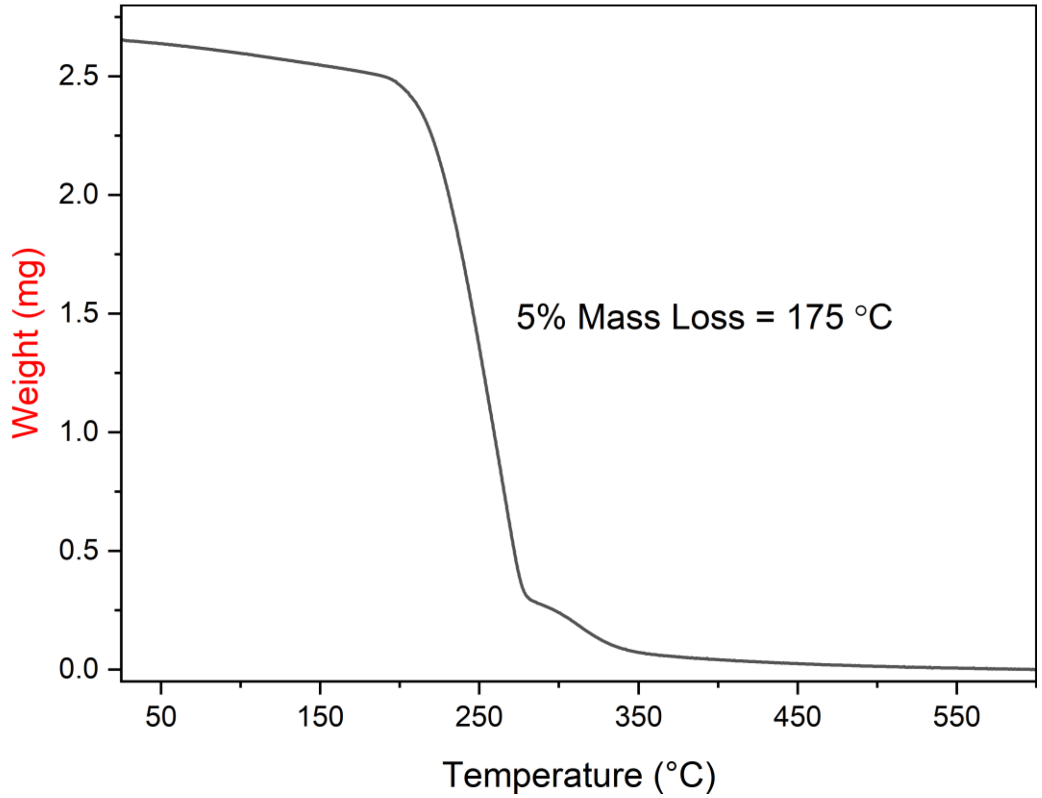

Figure S40. TGA of topochemical polymerization at 40 °C.

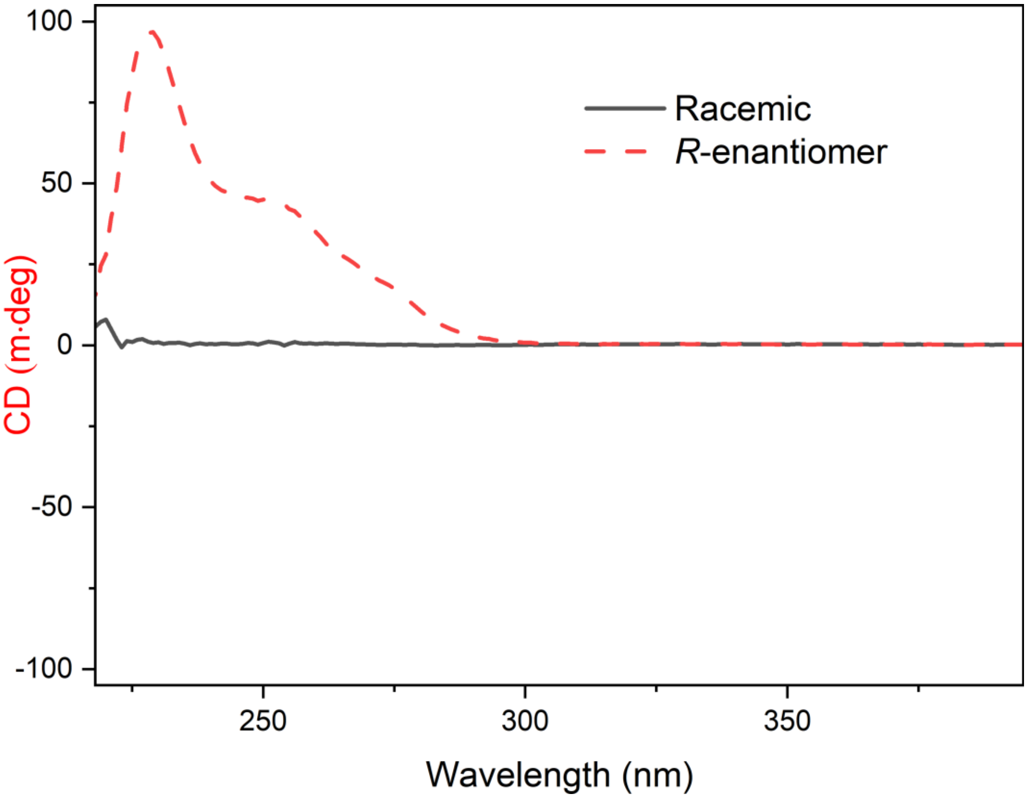

Figure S41. CD spectrum of racemic and enantiopure **POTT**.

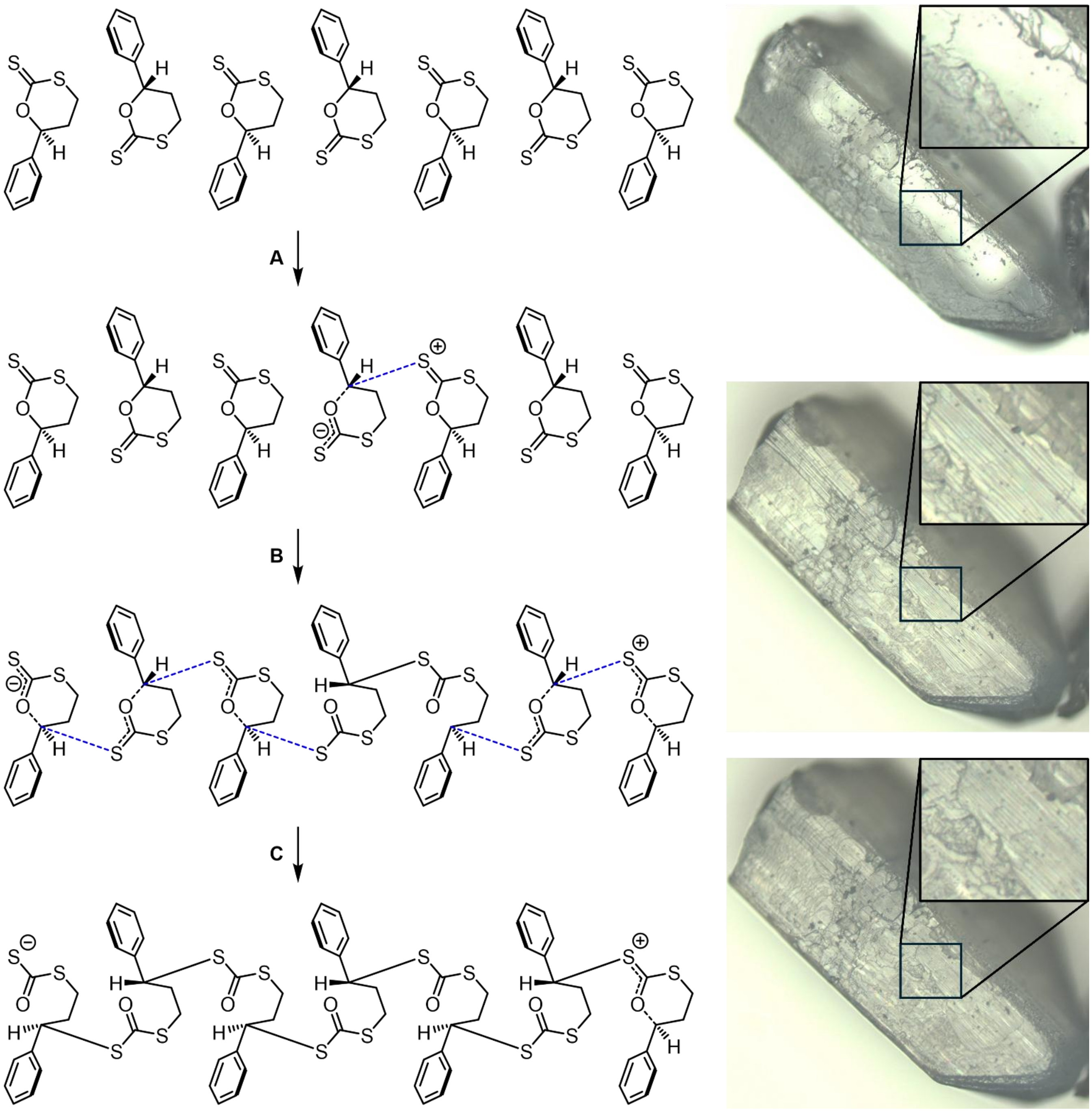

Figure S42. Proposed mechanism for the topochemical ring-opening of **OTT**.

References

1. Bruker, *SAINT*, *v8.37A*, Bruker AXS Inc., Madison, Wisconsin, USA.

2. Krause, L.; Herbst-Irmer, R.; Sheldrick, G.; Stalke, D. *J. Appl .Crystallogr*. **2015**, *48*, 3–10.

3. Sheldrick, G. *Acta Crystallogr. A*, **2015**, *71*, 3–8.

4. Dolomanov, O.; Bourhis, L.; Gildea, R.; Howard, J.; Puschmann, H. *J. Appl. Crystallogr*. **2009**, *42*, 339–341.

5. D. Kratzert, D. *FinalCif*, *V109*, https://dkratzert.de/finalcif.html.

6. Groom, C.R.; Bruno, I.J.; Lightfoot, M.P.; Ward, S.C. *Acta Cryst.* **2016**, *B72*, 171–179.

7. These analyses were performed using Mercury 4.0: from visualization to analysis, design and prediction. Macrae, C.F.; Sovago, I.; Cottrell, S.J.; Galek, P.T.A.; McCabe, P.; Pidcock, E.; Platings, M.; Shields, G.P.; Stevens, J.S.; Towler, M.; Wood, P.A. *J. Appl. Cryst.* **2020**, ***53***, 226-235.

8. Martín, J.; Stingelin, N.; Cangialosi, D. *J. Phys. Chem. Lett*. **2018**, *9*, 990-995.

9. Sommerville, P.J.W.; Li, Y.; Dong, B.X.; Zhang, Y.; Onorato, J.W.; Tatum, W.K.; Balzer, A.H.; Stingelin, N.; Patel, S.N.; Nealey, P.F.; Luscombe, C.K. *Macromolecules* **2020**, *53*, 7511-7518.

10. Walsh, D.J.; Lau, S.H.; Hyatt, M.G.; Guironnet, D. *J. Am. Chem. Soc.* **2017**, *139*, 13644-13647.

11. These torsion angles were calculated with the program CrystalMaker 11.
